# RAB-10 Promotes EHBP-1 Bridging of Filamentous Actin and Tubular Recycling Endosomes

**DOI:** 10.1371/journal.pgen.1006093

**Published:** 2016-06-06

**Authors:** Peixiang Wang, Hang Liu, Yu Wang, Ou Liu, Jing Zhang, Adenrele Gleason, Zhenrong Yang, Hui Wang, Anbing Shi, Barth D. Grant

**Affiliations:** 1 Department of Medical Genetics, School of Basic Medicine and the Collaborative Innovation Center for Brain Science, Tongji Medical College, Huazhong University of Science and Technology, Wuhan, Hubei, China; 2 Department of Molecular Biology and Biochemistry, Rutgers University, Piscataway, New Jersey, United States of America; 3 Institute for Brain Research, Huazhong University of Science and Technology, Wuhan, Hubei, China; 4 Key Laboratory of Neurological Disease of National Education Ministry, Tongji Medical College, Huazhong University of Science and Technology, Wuhan, Hubei, China; University of California San Francisco, UNITED STATES

## Abstract

EHBP-1 (Ehbp1) is a conserved regulator of endocytic recycling, acting as an effector of small GTPases including RAB-10 (Rab10). Here we present evidence that EHBP-1 associates with tubular endosomal phosphatidylinositol-4,5-bisphosphate [PI(4,5)P2] enriched membranes through an N-terminal C2-like (NT-C2) domain, and define residues within the NT-C2 domain that mediate membrane interaction. Furthermore, our results indicate that the EHBP-1 central calponin homology (CH) domain binds to actin microfilaments in a reaction that is stimulated by RAB-10(GTP). Loss of any aspect of this RAB-10/EHBP-1 system in the *C*. *elegans* intestinal epithelium leads to retention of basolateral recycling cargo in endosomes that have lost their normal tubular endosomal network (TEN) organization. We propose a mechanism whereby RAB-10 promotes the ability of endosome-bound EHBP-1 to also bind to the actin cytoskeleton, thereby promoting endosomal tubulation.

## Author Summary

Endosomes are intracellular organelles that sort protein and lipid components integral to the membrane, as well as more loosely associated lumenal content, for delivery to distinct intracellular destinations. Endosomes associated with recycling cargo back to the plasma membrane are often tubular in morphology, and this morphology is thought to be essential for recycling function. Our previous work identified a particularly dramatic network of endosomal tubules involved in membrane protein recycling in the basolateral intestinal epithelial cells of *C*. *elegans*. Our subsequent genetic analysis of basolateral recycling in this system identified a number of key regulators of these endosomes, including the small GTPase RAB-10 and its effector EHBP-1. Our new work presented here shows that EHBP-1 promotes endosomal tubulation by linking the membrane lipid PI(4,5)P2 to the actin cytoskeleton, and that the linkage of EHBP-1 to actin is enhanced by the interaction of EHBP-1 with RAB-10. This work has broad implications for how endosomal tubulation occurs in all cells, and has specific implications for the role of EHBP-1 in related processes such as insulin-stimulated recycling of glucose transporters in human adipocytes, a process intimately linked to type II diabetes.

## Introduction

Transmembrane proteins enter cells via several endocytic pathways including clathrin-dependent endocytosis (CDE) and a variety of less well understood clathrin-independent endocytosis (CIE) mechanisms [1–3]. After internalization some receptors will be recycled back to the plasma membrane via the endocytic recycling compartment (ERC) [4, 5]. Recycling endosome transport is known to be essential for diverse biological processes, including cell migration, cytokinesis, and synaptic plasticity [5].

In the *C*. *elegans* intestine the small GTPase RAB-10 resides on a subset of basolateral endosomes where it regulates basolateral cargo recycling upstream of RME-1/EHD, a membrane remodeling protein with Dynamin-like features [6–9]. While the cargo-specificity of RME-1 is broad, RAB-10 appears more specific, with especially potent effects on the recycling of transmembrane proteins internalized by CIE, such as the model CIE cargo hTAC (the alpha-chain of the human IL2 receptor) [6, 10]. Rab10 function in mammalian cells appears highly conserved, where Rab10 is highly enriched on the membranes of the common recycling endosomes and regulates basolateral recycling in polarized epithelial cells [11]. Likewise, in mammalian adipocytes, Rab10 functions in the insulin-stimulated recycling of glucose transporter GLUT4 [12]. The calponin homology (CH) domain protein Ehbp1 has also been reported to function in GLUT4 recycling in adipocytes, associated with the RME-1 homologs EHD1 and EHD2 [13, 14].

In our previous work we determined that *C*. *elegans* EHBP-1 binds to the GTP-loaded conformation of RAB-10 through its C-terminal domain (a predicted coiled-coil) and functions with RAB-10 in the intestinal basolateral recycling of hTAC, and in the neuronal recycling of AMPA-type glutamate receptor GLR-1 [10, 15]. EHBP-1 labels an extensive network of tubular endosomes in the intestine where it colocalizes with recycling cargo, and is also found on connected punctate endosomal membranes where it colocalizes with RAB-10. Loss of EHBP-1 produces phenotypes that strongly resemble those produced upon loss of RAB-10. These include RAB-10-specific phenotypes in polarized cells such as the intestinal epithelium, including accumulation of enlarged basolateral endosomes filled with fluid-phase markers and hTAC, and the abnormal accumulation of endosomal GLR-1 in interneurons [10, 15]. *ehbp-1* mutants or RNAi also produce phenotypes in non-polarized cells very similar to simultaneous loss of RAB-10 and its closest paralog RAB-8, including variable larval arrest, and fully penetrant adult sterility due to a failure in germline membrane transport and oocyte growth [15]. In Drosophila dEHBP1 has also been reported to act with Rab11 [16, 17].

Our previous studies found that a truncated form of EHBP-1 lacking the RAB-10 interaction domain remained membrane associated, raising the question of how EHBP-1 associates with endosomal membranes [15]. Although not apparent in simple homology searches, a purely computational study using sequence profile searches with profile–profile comparison and fold recognition methods classified the EHBP-1 N-terminus as a putative C2-like domain (NT-C2) that could potentially mediate direct membrane binding [18]. It has been shown that endosomal recruitment of some conserved recycling regulators depends on the regulatory lipid phosphatidylinositol-4,5-bisphosphate [PI(4,5)P2] [9]. PI(4,5)P2 is enriched at the plasma membrane and recycling endosomes, and membrane bending proteins associated with recycling function such as RME-1/EHD and AMPH-1/Amphiphysin/BIN1 have been shown to associate with membrane structures enriched in PI(4,5)P2 [9, 19, 20]. In fact, we have previously shown that the PI(4,5)P2 level in basolateral recycling endosomes is modulated by RAB-10, in part through its effector CNT-1, an ARF-6 GAP [20]. Other reports also indicate a requirement for phosphatidylinositol-4-phosphate (PI4P) in recycling endosome function [21]. These findings imply that EHBP-1 could be targeted to recycling endosomes via PI(4,5)P2 and/or PI(4)P binding.

In addition to its N-terminal C2-like and C-terminal RAB-10-binding domains, EHBP-1 harbors a central CH domain. CH domains in different proteins are known to bind to the cytoskeleton, but vary in their specificity, with some binding to the microtubule cytoskeleton and others binding to actin filaments [22]. Requirements for the microtubule and actin cytoskeletons are well established in the endosomal system [23–26].

The actin cytoskeleton also plays essential roles along the endocytic pathway. First identified in studies of endocytosis in yeast, Arp2/3-dependent nucleation of actin at endocytic sites has been observed in many organisms, including mammals, and is thought to contribute to membrane fission [27–31]. Furthermore, certain forms of endocytic recycling are also actin-dependent. For instance, actin depolymerization results in the retention of TAC in tubular recycling endosomes together with Arf6, suggesting the necessity of actin function in Arf6-mediated recycling transport [32, 33]. Retromer/WASH mediated local actin polymerization on endosomes has alternately been reported to enhance the fission of tubular cargo carriers from endosomes, or to stabilize tubular extensions for cargo loading prior to their release by fission [34, 35].

Cargo carriers in the endosomal system are often tubular in nature, and their tubular shape has been proposed to help sort membrane intrinsic components away from lumenal content [4, 36]. The endocytic recycling compartment in mammals is composed of a dense collection of endosomal tubules and vesicles [4]. In the *C*. *elegans* intestine the basolateral recycling compartment enriched in CIE cargo, EHBP-1, and RME-1, is highly tubular in nature and appears to have many interconnections [8, 15, 37]. The entire network collapses to vesicles upon loss of RAB-10 or EHBP-1, suggesting that they contribute to the formation and/or maintenance of such tubular endosomes [6, 15].

To further dissect the function of RAB-10 effector EHBP-1, we studied individual domains of EHBP-1 *in vitro* and *in vivo*, and characterized EHBP-1 regulation by RAB-10. Here we demonstrate that the NT-C2 and CH domains are both indispensible for proper EHBP-1 function. We found that the EHBP-1 NT-C2 domain has an intrinsic ability to associate with endosomal membranes. RNAi-mediated knockdown of phosphoinositide kinases, colocalization assays with PI(4,5)P2 biosensor PH(PLCδ)-GFP, and liposome co-sedimentation assays revealed that the EHBP-1 NT-C2 domain preferentially associates with PI(4,5)P2 enriched endosomes via predicted patches of basic residues within the NT-C2 domain. Our biochemical studies indicate that the EHBP-1 CH domain preferentially binds to actin filaments and not microtubules, and EHBP-1 colocalizes with endosomal actin *in vivo*. Remarkably we find that the interaction of the EHBP-1 C-terminal domain with RAB-10(GTP) enhances the actin filament affinity of EHBP-1 via its central CH domain. Our data demonstrates that RAB-10 regulates EHBP-1 actin binding and suggests that RAB-10 and EHBP-1 function together with actin to create and/or maintain endosomal tubulation.

## Results

### The NT-C2 domain is required for EHBP-1 function and localization

**Fig 1:**
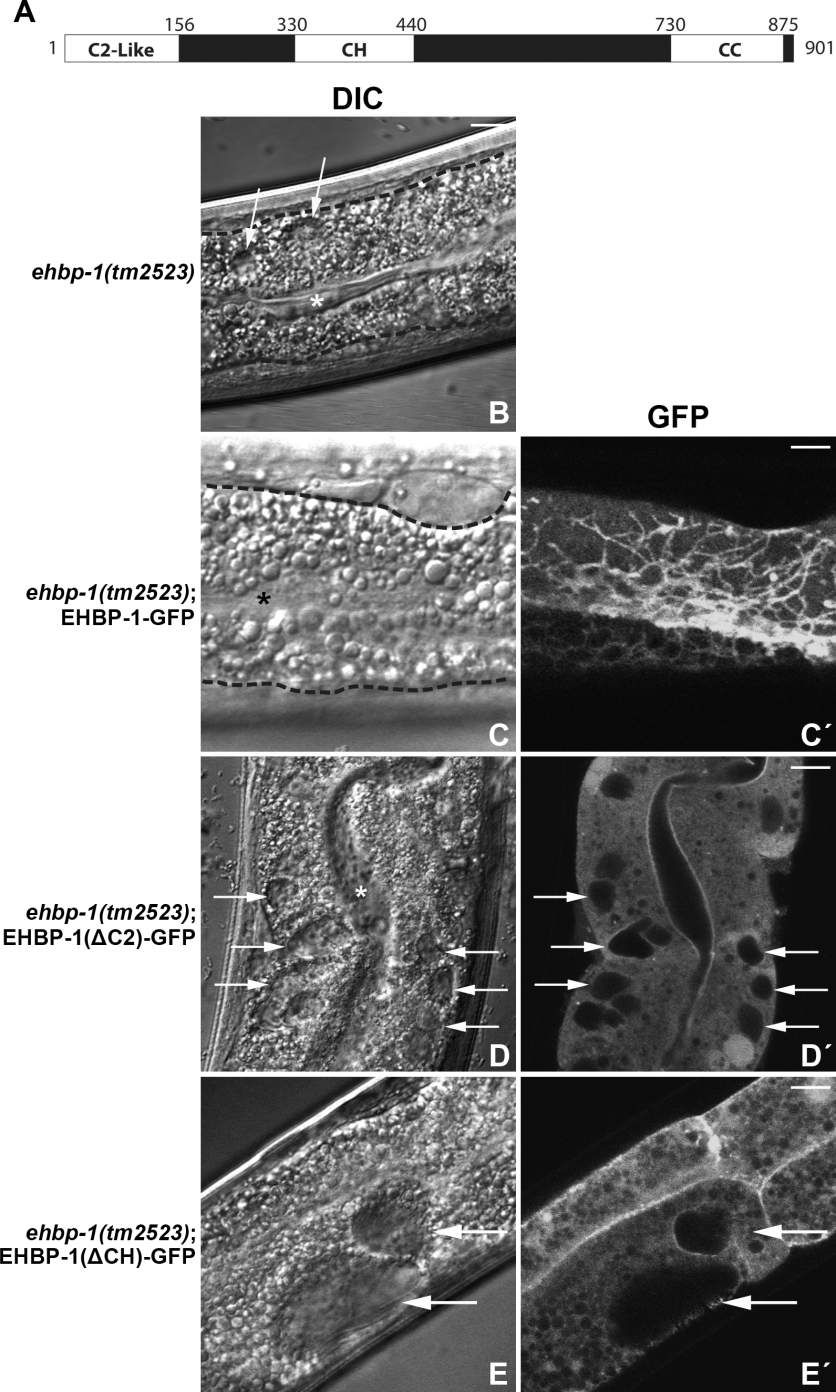
EHBP-1 lacking the N-terminal C2-like or central CH domain failed to rescue the vacuolated phenotype in the *C*. *elegans* intestine. **(A)** Schematic representation of EHBP-1 sequence regions including the N-terminal C2-like domain (NT-C2), internal CH (Calponin Homology) domain and C-terminal coiled-coil (CC) domain. Protein domains are displayed as white boxes and amino acid numbers are indicated. **(B)** In *ehbp-1(tm2523)* deletion mutant intestinal cells, abnormally enlarged vacuoles can be observed by DIC microscopy. **(C-C')** The vacuolated intestine phenotype can be fully rescued by expression of EHBP-1-GFP. **(D-D')** Many large vacuoles can still be observed in animals expressing EHBP-1 lacking the NT-C2 domain. **(E-E')** Expression of EHBP-1 lacking the CH domain failed to rescue the vacuolated intestine phenotype. Tissue boundaries of intestines are outlined in **(B and C)**. Arrows indicate vacuolated structures in the intestinal cells. Asterisks indicate intestinal lumens. Scale bars represent 10 μm.

In *ehbp-1(tm2523)* deletion mutants, or upon RNAi of *ehbp-1*, the tubular endosomal network is disrupted and large vacuoles accumulate near the basolateral membranes of the intestinal cells, a phenotype very similar to *rab-10* mutants. Such vacuoles are grossly enlarged early endosomes that can be labeled by fluid-phase endocytosis markers taken up from the basolateral surface (Fig 1B, S1A and S1A' and S1E Fig) [15]. This vacuole phenotype can be fully rescued by intestine-specific expression of tagged forms of full-length EHBP-1 (Fig 1C and 1C', S1B–S1B'' and S1E Fig).

EHBP-1 contains three distinct protein domains, including an N-terminal C2-like domain (NT-C2), central CH (Calponin Homology) domain, and C-terminal predicted coiled-coil (CC) domain (Fig 1A) [15, 18]. Our previous studies showed that the predicted CC domain of EHBP-1 binds to RAB-10(GTP), and EHBP-1 missing the CC domain does not rescue the *ehbp-1* mutant intestinal vacuole phenotype [15]. However, removal of the RAB-10-binding CC-domain does not cause redistribution of EHBP-1 to the cytoplasm. Rather, EHBP-1 lacking the CC-domain remains associated with misshapen endosomal membranes and acts as a dominant negative, impairing recycling [15]. These results indicated that while the CC-domain is important for function, EHBP-1 must have a mechanism for membrane association independent of the RAB-10 binding domain.

Bioinformatics analysis suggests that the N-terminus of EHBP-1 contains a C2-like domain termed the NT-C2. Since many C2 domains bind directly to membrane lipids, the newly proposed NT-C2 domain is an excellent candidate to mediate such EHBP-1 membrane binding [18]. To better understand the functional significance of the predicted EHBP-1 NT-C2 domain, we analyzed the ability of GFP-tagged EHBP-1 missing the predicted NT-C2 domain (EHBP-1(ΔNT-C2)-GFP) to rescue the intestinal vacuolation of *ehbp-1(tm2523)* mutants. Intestinally expressed EHBP-1(ΔNT-C2)-GFP failed to rescue (Fig 1D and 1D', S1C–S1C'' and S1E Fig), and was much more diffusive than intact EHBP-1, consistent with a function for the NT-C2 domain in recruiting EHBP-1 to membranes. Furthermore, intestinal over-expression of the CH-CC fragment (EHBP-1(ΔNT-C2)) in an otherwise wild-type background disrupted recycling cargo hTAC-GFP tubular endosomal localization and caused hTAC-GFP accumulation on enlarged endosomes and vacuoles (S2E–S2F' and S2H and S2I Fig). In the absence of the NT-C2 domain, the only remaining localized EHBP-1 signal was restricted to small punctate structures (S2A Fig), unlike full length EHBP-1-GFP, which localizes strongly to abundant tubular endosomes in the basolateral cortex, as well as apparently attached endosomal puncta (Fig 1C').

Notably, the residual EHBP-1(ΔNT-C2)-GFP labeled puncta were lost upon removal of RAB-10 (S2B Fig). Taken together these results suggest that the NT-C2 domain of EHBP-1 is important for EHBP-1 function and the recruitment to tubular endosomal membranes, with a contribution by the RAB-10-binding CC-domain in recruitment to punctate endosomes.

### The EHBP-1 NT-C2 domain preferentially associates with endosomal PI(4,5)P2

**Fig 2:**
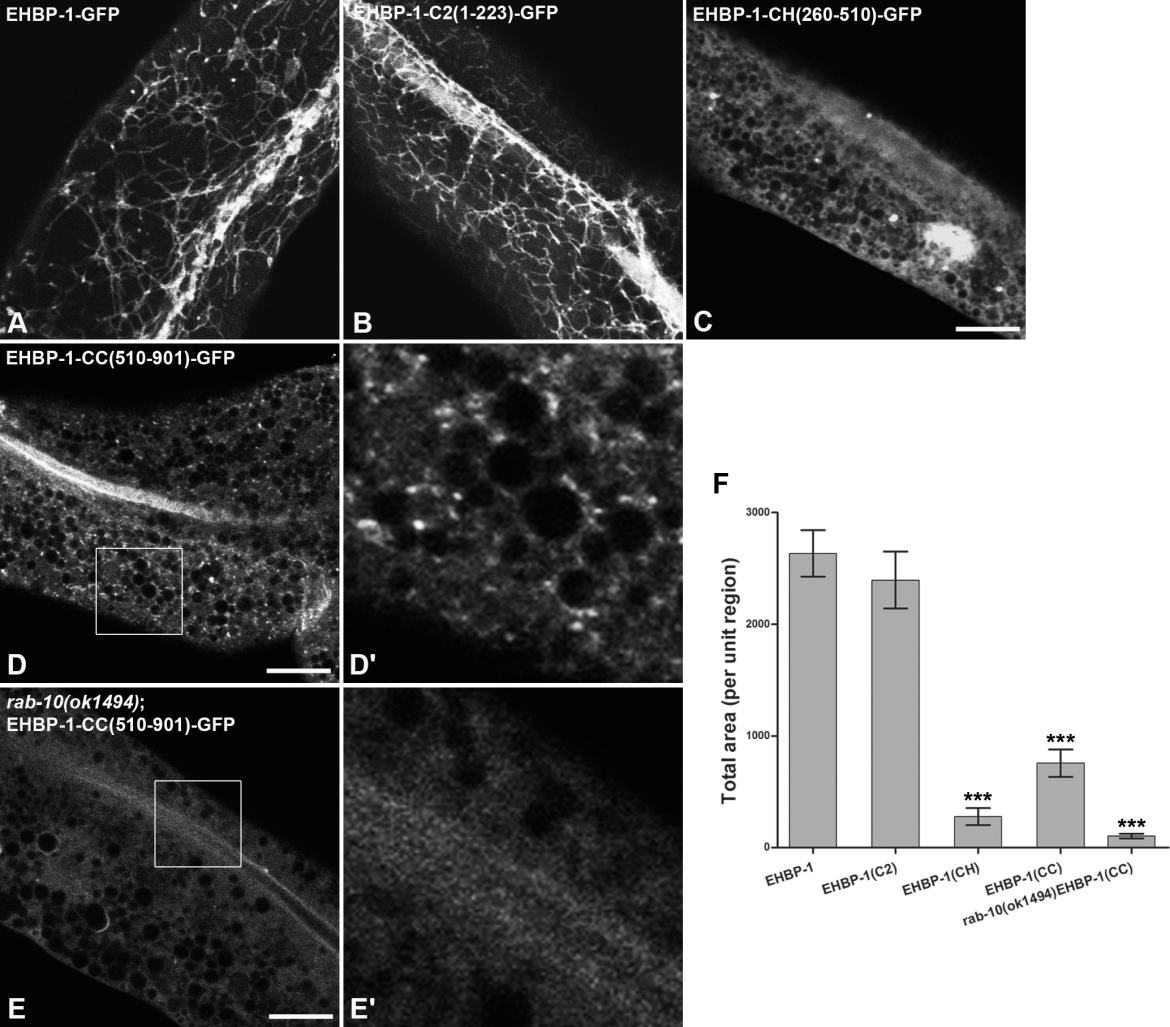
Analysis of individual proteins domains from EHBP-1 for sufficiency in subcellular localization. Representative confocal images are shown for EHBP-1-GFP, EHBP-1-NT-C2(aa1-223)-GFP, EHBP-1-CH(aa260-510)-GFP, EHBP-1-CC(aa510-901)-GFP in wild-type animals, and EHBP-1-CC(aa510-901)-GFP in the *rab-10(ok1494)* mutant. **(A)** EHBP-1 labels intestinal basolateral punctate and tubular endosomal structures. **(B)** The EHBP-1 NT-C2 domain localizes to tubular and punctate membrane structures similar to those labeled by full length EHBP-1. **(C)** The EHBP-1 CH domain localized diffusely in the intestinal cells with sparse puncta visible above background. **(D-D')** The EHBP-1 CC domain localized to punctate endosomes near the basolateral membrane. **(E-E')** The EHBP-1 CC domain appeared diffusive in *rab-10(ok1494)* mutant animals. Enlarged images (4x) of boxed regions are shown in the insets. Total fluorescence area of GFP signal within unit regions was quantified in **(F)**. Error bars are SEM (n = 18 each, 6 animals of each genotype sampled in three different unit regions of each intestine defined by a 100 x 100 (pixel^2^) box positioned at random). Asterisks indicate significant differences in the one-tailed Student’s t-test (*** p< 0.001). Scale bars represent 10 μm.

**Fig 3:**
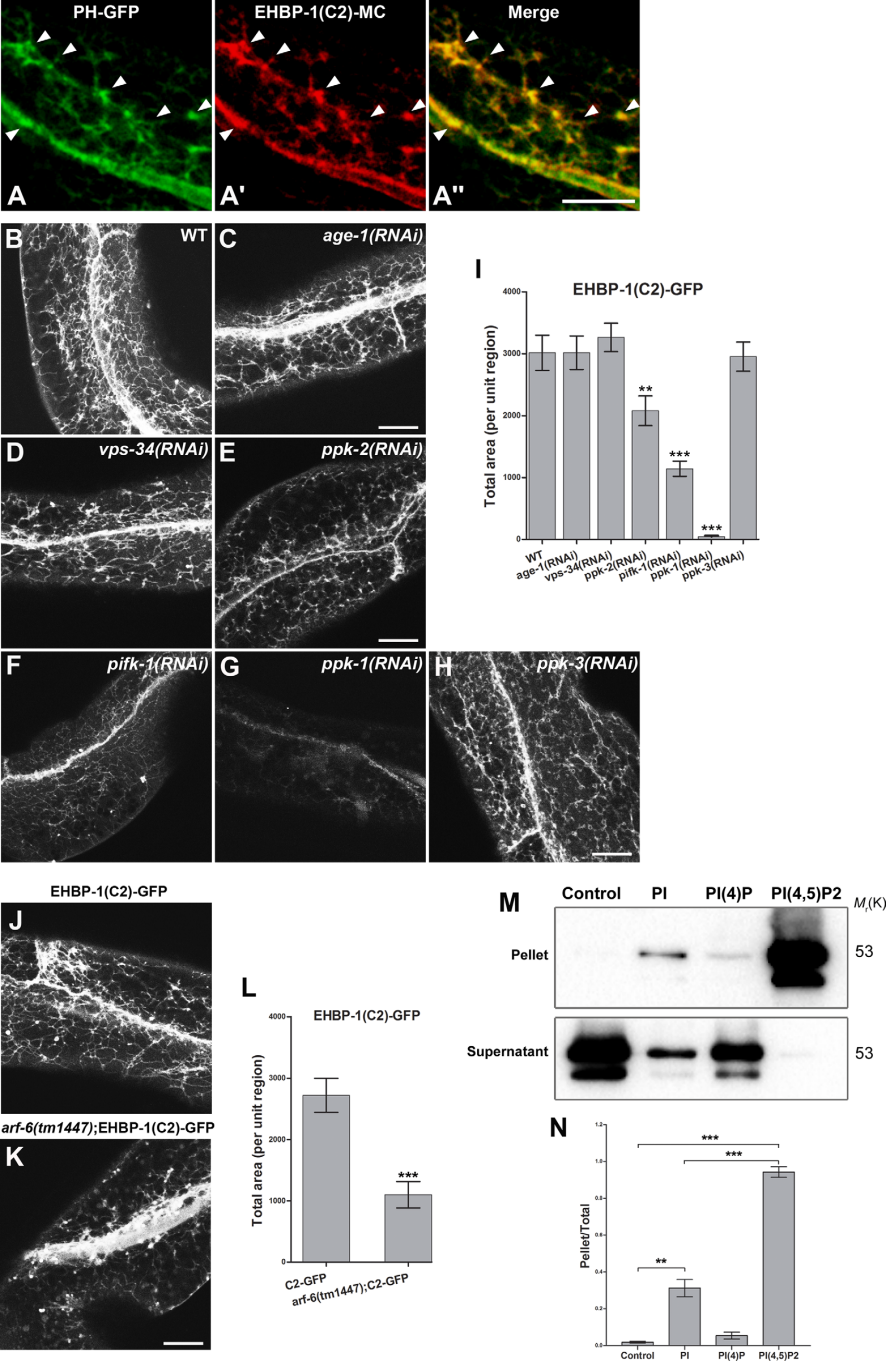
The EHBP-1 NT-C2 domain requires PI(4,5)P2 for localization to endosomal structures. **(A-A")** EHBP-1(NT-C2 domain)-mCherry colocalizes with PH(PLC δ)-GFP (PI(4,5)P2 biosensor) on tubular and punctate endosomes. Representative images from confocal image stacks are shown. All images were acquired in intact living animals expressing GFP and mCherry tagged proteins specifically in intestinal epithelial cells. Arrowheads indicate structures labeled by both PH-GFP and EHBP-1(NT-C2)-mCherry. **(B-I)** Representative confocal images are shown for the EHBP-1(NT-C2 domain)-GFP labeling pattern in animals after RNAi-mediated depletion of PI-kinases involved in phosphatidylinositol metabolism. **(B)** Wild-type EHBP-1(NT-C2)-GFP localized to basolateral punctate and tubular endosomal structures in intestinal cells. **(C-D)** EHBP-1(NT-C2)-GFP subcellular localization was not affected upon knockdown of PI(4,5)P2-directed PI3-kinase AGE-1 and PI-directed PI3-kinase VPS-34. **(E-F)** EHBP-1(NT-C2)-GFP punctate and tubular endosomal labeling decreased (~62%) after RNAi knockdown of PI-directed PI4-kinase PIFK-1, while the knockdown of PI(5)P-directed PI4-kinase PPK-2 caused a moderate EHBP-1(NT-C2)-GFP labeling decrease (~35%). **(G-H)** EHBP-1(NT-C2)-GFP lost its endosomal labeling (~98%) in *ppk-1(RNAi)* animals (PI(4)P-directed PI5-kinase). Knockdown of PI(3)P-directed PI5-kinase PPK-3 did not affect EHBP-1(NT-C2)-GFP labeling on tubular endosomes. Total fluorescence area of GFP signal within unit regions was quantified in **(I)**. **(J-K)** EHBP-1(NT-C2)-GFP labeling on basolateral tubules and puncta decreased in *arf-6* mutants (~65%), total fluorescence area of EHBP-1(NT-C2)-GFP labeled structures within unit regions was quantified in **(L)**. Error bars are SEM (n = 18 each, 6 animals of each genotype sampled in three different regions of each intestine defined by a 100 x 100 (pixel^2^) box positioned at random). Asterisks indicate significant differences in the one-tailed Student’s t-test (**p< 0.01, *** p< 0.001). Scale bars represent 10 μm. **(M)** Western blotting of supernatant and pellet fractions from liposome co-sedimentation assays. Binding reactions were performed in the presence of liposomes containing 0% PI (Control), 5% PI, 5% PI(4)P or 5% PI(4,5)P2. Liposomes were incubated with 3ug GST-EHBP-1(NT-C2) as indicated. **(N)** Band intensities in pellet and supernatant blots were quantified, Pellet/Total ratio of each sample was calculated (Pellet/Total = pellet band intensity/pellet band intensity + supernatant band intensity). Error bars are SEM (n = 3). Asterisks indicate significant differences in the one-tailed Student’s t-test (**p< 0.01, *** p< 0.001).

Next we asked if the NT-C2 domain of EHBP-1 is sufficient to direct GFP to endosomal structures. When expressed in the *C*. *elegans* intestine, EHBP-1-NT-C2(aa1-223)-GFP localized to tubular and punctate endosomes, very similar to full length EHBP-1-GFP (Fig 2A and 2B and Fig 2F), co-localizing with PI(4,5)P2 biosensor PH(PLCδ)-GFP on basolateral tubular and punctate membrane structures (Fig 3A–3A") [15]. Like full length EHBP-1, EHBP-1-NT-C2(aa1-223)-GFP also overlapped with ARF-6-RFP and RFP-RAB-10 on basolateral puncta (S3A–S3B'' Fig). EHBP-1-NT-C2(aa1-223)-GFP showed little colocalization with RFP-2xFYVE, a marker for early endosome enriched lipid PI(3)P (S4A–S4B'' Fig). Together our results indicate that the EHBP-1 NT-C2 domain is sufficient to direct EHBP-1 to endosomes, probably via direct membrane binding.

Since tubular recycling endosomes are enriched in PI(4,5)P2 and the EHBP-1 NT-C2 domain displayed a similar subcellular distribution to a PI(4,5)P2 biosensor, we tested whether PI(4,5)P2 or other phosphoinositides are important for NT-C2 membrane recruitment, using RNAi-based knockdown of PI-kinases involved in phosphatidylinositol metabolism (Fig 3B–3I). Loss of a key EHBP-1 binding lipid is expected to result in diffusion of NT-C2-GFP protein *in vivo*. Depletion of PI-directed PI4-kinase PIFK-1 produced a nearly 3-fold decrease in EHBP-1(NT-C2)-GFP labeling of endosomal puncta and tubules (Fig 3F and Fig 3I). RNAi knockdown of PI(5)P-directed PI4-kinase PPK-2 produced a more modest (~35%) decrease in EHBP-1(NT-C2)-GFP endosome association (Fig 3E and Fig 3I). PI(4,5)P2 levels are also regulated by PI5-kinases. Consistent with PI(4,5)P2 being a key lipid in EHBP-1 membrane recruitment, we observed a strong decrease in EHBP-1(NT-C2)-GFP labeling of endosomal puncta and tubules after RNAi-mediated depletion of PI(4)P-directed PI5-kinase PPK-1, with a ~98% decrease in average intensity (Fig 3G and Fig 3I). Knockdown of PI(3)P-directed PI5-kinase PPK-3 did not affect EHBP-1(NT-C2)-GFP distribution (Fig 3H and Fig 3I). In addition we did not find any effects on EHBP-1(NT-C2)-GFP localization after RNAi of PI3-kinases including PI(4,5)P2-directed PI3-kinase AGE-1 and PI-directed PI3-kinase VPS-34 (Fig 3C and 3D and Fig 3I). Taken together, these results suggested that EHBP-1 membrane recruitment requires PI(4,5)P2 in endosomes, whose level is mainly regulated by PI5-kinase PPK-1 and PI4-kinase PIFK-1, and to a lesser extent PI4-kinase PPK-2. Indeed, similar to the *ehbp-1(tm2523)* mutant phenotype, hTAC-GFP lost its tubular endosomal localization and accumulated in punctate structures upon the knockdown of PPK-1, suggesting that endosomal PI(4,5)P2 is required for the recycling function of EHBP-1 (S2G–S2G' Fig).

PI(4,5)P2 levels on endosomes is regulated by GTPase ARF-6, presumably through activation of PI5-kinase PPK-1 [20]. Therefore, EHBP-1 recruitment to endosomes may be dependent on ARF-6. Consistent with the action of ARF-6 in PPK-1 regulation, we found that EHBP-1(NT-C2)-GFP basolateral tubular and puncta labeling decreased in *arf-6* mutants (~60%) (Fig 3J–3L).

To further define the endosomal phosphoinositide association preference of EHBP-1, we tested the interaction of purified recombinant GST-NT-C2 with liposomes in sedimentation assays. We found that GST-NT-C2 preferentially pelleted with liposomes containing 5% PI(4,5)P2, with much less co-sedimentation with liposomes containing PI or PI(4)P (Fig 3M and 3N and S4C Fig).

### Basic amino acid patches are required for NT-C2-domain recruitment to membranes

**Fig 4:**
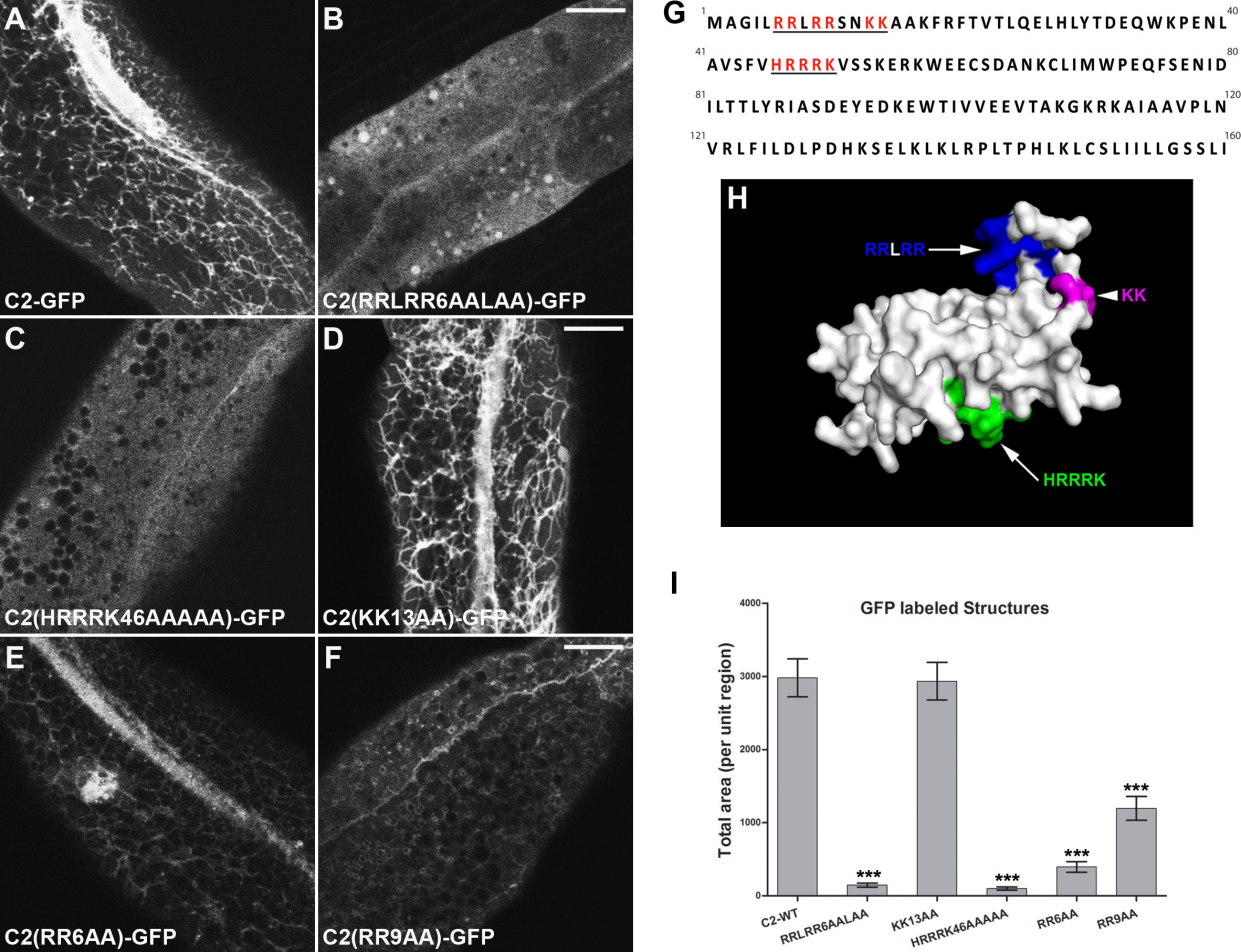
Basic residue patches RRLRR and HRRRK are both required for membrane association of EHBP-1 NT-C2 domain. **(A)** Wild-type EHBP-1(NT-C2)-GFP labels punctate and tubular endosomal structures. **(B)** Compared with the wild-type NT-C2 domain, EHBP-1(NT-C2:RRLRR6AALAA)-GFP lost endosomal association and appeared diffusive in the cytosol (~95% decrease in structures labeling). **(C)** Alanine substitutions of HRRRK caused cytosolic diffusion of NT-C2-GFP (~97% decrease in structure labeling). **(D)** EHBP-1(NT-C2:KK13AA)-GFP subcellular localization was not affected. **(E-F)** Loss of either RR6 or RR9 decreased labeling of EHBP-1(NT-C2)-GFP on endosomal structures (~87% and ~60% decrease respectively). **(G)** Annotated amino acid sequence of EHBP-1 NT-C2 domain (aa1-160). Basic residues are indicated by red, and the clusters are underlined. **(H)** Surface diagram of the EHBP-1 N-terminal (aa1-160) homology model (created via HHpred and modeler). RRLRR, indicated by blue, is located at the extreme N-terminus, contributing to a concavity formation together with surrounding hydrophobic residues. HRRRK, indicated by green, is located at the other side of the model surface. **(I)** Total area (per unit region) was quantified for GFP-labeled structures in **(A-F)**. Error bars are SEM (n = 18 each, 6 animals of each genotype sampled in three different regions of each intestine defined by a 100 x 100 (pixel^2^) box positioned at random). Asterisks indicate significant differences in the one-tailed Student’s t-test (*** p< 0.001). Scale bars represent 10 μm.

To determine residues that may contribute to membrane association, we analyzed evolutionary sequence conservation within the NT-C2 domain, and mapped conserved positively charged residues onto a homology model that we constructed. Our model suggests that the N-terminal 160 amino acids of EHBP-1 folds into a globular domain consisting of seven β-strands and an α-helical segment between strand-5 and strand-6. A patch of basic residues at the extreme N-terminus of the fold prior to strand-1 is predicted in this model to contribute to the formation of a concavity in the β sheet, on the “upper surface” comprised of a constellation of basic and hydrophobic residues (Fig 4H).

We performed alanine substitution in three areas of the NT-C2 domain in the context of the NT-C2-GFP intestinal expression construct (Fig 4G). Compared with wild type, modification of the four arginines within the patch prior to strand-1, predicted to line the concavity, results in loss of association with tubular endosomal membranes and diffusion of the mutated NT-C2-GFP within the cytosol (Fig 4A and 4B and Fig 4I). Mutation of pairs of arginines within this sequence reduced but did not eliminate membrane association, suggesting that all four arginines contribute to NT-C2 domain membrane binding (Fig 4E and 4F and Fig 4I). Furthermore, we assayed the vacuole phenotype in *ehbp-1(tm2523)* mutants animals expressing EHBP-1(RRLRR6AALAA)-GFP. In contrast to WT EHBP-1 (S1B–S1B'' Fig), the number and size of vacuoles were not rescued by EHBP-1(RRLRR6AALAA)-GFP, indicating that the RRLRR motif is critical for EHBP-1 recycling function (S4D and S4E Fig). Mutation of nearby lysines, predicted to face away from the cleft, had no effect on the association of NT-C2-GFP with tubular endosomes (Fig 4D and Fig 4I). Another patch of basic residues (HRRRK of strand-2) on the predicted surface of the NT-C2 domain also appears to contribute to NT-C2 membrane association, as mutation of this sequence also produced a diffusive localization (Fig 4C and Fig 4I). Collectively, our results suggest that EHBP-1 NT-C2 domain associates with PI(4,5)P2 enriched endosomal membranes through two patches of basic amino acids.

### The CH-domain is required for EHBP-1 function but not subcellular localization

To determine if the CH domain is important for the subcellular localization of EHBP-1 to endosomes we expressed GFP-tagged EHBP-1 lacking the CH domain (EHBP-1(ΔCH)-GFP) in the intestinal epithelia. The CH domain did not appear to be a major determinant of localization, since EHBP-1(ΔCH)-GFP was enriched on tubular and punctate membranes in a pattern indistinguishable from intact EHBP-1-GFP (S2C Fig). This localization was also RAB-10-dependent like full-length EHBP-1, displaying significant intracellular accumulation on puncta and vacuoles in *rab-10(ok1494)* mutant animals (S2D Fig). Nevertheless, intestinally expressed EHBP-1(ΔCH)-GFP failed to rescue the *ehbp-1(tm2523)* mutant vacuole phenotype (Fig 1E–1E', S1D–S1D'' and S1E Fig), indicating that the CH domain is also indispensible for EHBP-1 function.

Unlike the NT-C2 domain above, which conferred robust localization to endosomes on its own, expression of the EHBP-1 CH-domain (aa260-510), fused to GFP, localized relatively diffusely in the intestinal cells. Sparse puncta were visible above background. These puncta partially overlapped with ARF-6 and RAB-10, indicating that they represent very weak recruitment to endosomes (Fig 2C and Fig 2F and S3C–S3D'' Fig).

By contrast, the C-terminal RAB-10-binding domain, expressed as EHBP-1-CC(510-901aa)-GFP, lacked tubular localization but retained visible localization on punctate endosomes labeled by ARF-6-RFP and RFP-RAB-10 (Fig 2D and 2D' and Fig 2F and S3E–S3F'' Fig). RAB-10 was required for this punctate recruitment, since the punctate labeling of EHBP-1-CC-GFP was lost in *rab-10(ok1494)* mutant animals (Fig 2E and 2E' and Fig 2F). This is distinct from the full length EHBP-1 protein that remains membrane associated in a *rab-10* mutant background, presumably through the NT-C2 domain [15].

### The EHBP-1 CH domain preferentially interacts with actin microfilaments

CH domains have the potential to interact with cytoskeletal elements [22]. Structural studies on the kinetochore attached Ndc80 complex indicated that a CH-domain pair is involved in microtubule binding [38]. Mammalian EHBP1 was shown to colocalize with the cortical actin cytoskeleton in COS-1 cells, and overexpression of HA-EHBP1 induced cortical actin rearrangement [13]. Homology analysis suggests that the CH domain of *C*. *elegans* EHBP-1 most closely resembles the CH domain of β spectrin and the second CH domain of utrophin [39, 40].

**Fig 5:**
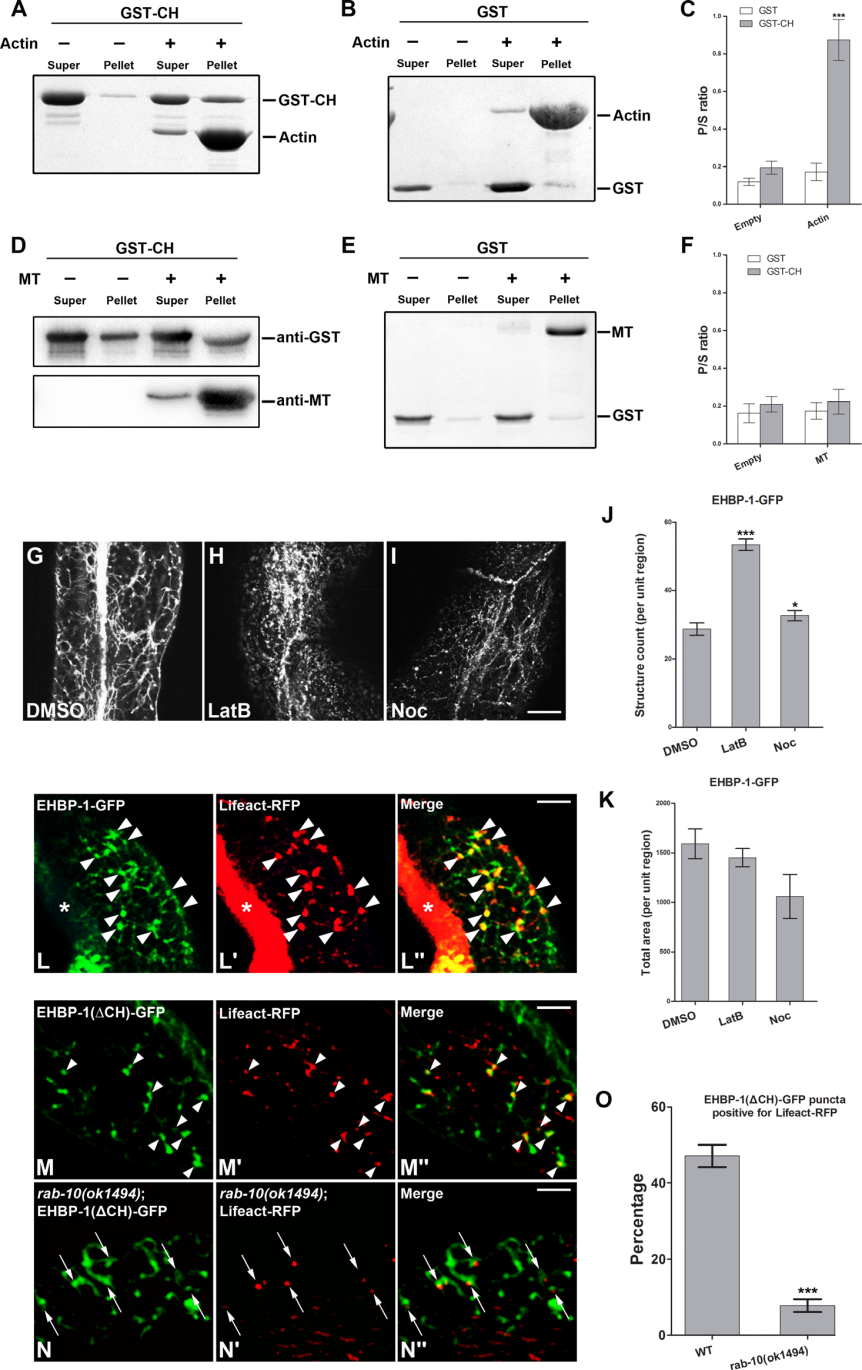
Interaction of the EHBP-1 CH domain with F-actin. **(A-B) The EHBP-1 CH domain co-sediments with actin filaments *in vitro*.** GST-CH sedimentation P/S ratio (pellet/supernatant) shifted from ~16% to ~80% with addition of actin filaments (coomassie blue stained gel). P/S ratio was quantified for GST-CH and GST-only in **(C)**, error bars are SEM (n = 3). Asterisks indicate significant differences in the one-tailed Student’s t-test (*** p< 0.001). **(D-E)** Compared with the control, microtubule addition did not enhance the sedimentation of GST-CH (anti-GST western blot). P/S ratio was quantified for GST-CH and GST-only in **(F)**, error bars are SEM (n = 3). **(G-K)** Integrity of EHBP-1 positive tubular endosomes requires intact F-actin and microtubule cytoskeletons. **(G)** EHBP-1-GFP mainly localized to normal appearing tubular endosomes after injection of control DMSO. **(H)** The intestinal EHBP-1-GFP positive tubular meshwork was disrupted, and EHBP-1-GFP puncta number increased by about 2-fold, after LatB treatment. **(I)** Microtubule-depolymerizing drug nocodazole (Noc) treatment did not fully disrupt EHBP-1 labeled tubular network. **(J-K)** EHBP-1-GFP labeled puncta number (structure count) and total fluorescence area (total area) of these puncta within unit region were quantified respectively. Error bars are SEM (n = 18, 6 animals of each treatment were sampled in three different unit regions of each intestine defined by a 100 x 100 (pixel^2^) box positioned at random). Asterisks indicate significant differences in the one-tailed Student’s t-test (*p< 0.05, *** p< 0.001). **(L-L")** Actin marker Lifeact-RFP colocalizes well with EHBP-1-GFP on basolateral punctate endosomes in intestinal cells. **(M-N" and O)** The overlap between Lifeact-RFP and EHBP-1-GFP requires the CH domain. EHBP-1(ΔNT-C2)-GFP colocalizes with actin marker Lifeact-RFP on punctate structures, however, loss of the EHBP-1 CH domain in a *rab-10* mutant background resulted in the decrease of EHBP-1-GFP and Lifeact-RFP overlap percentage (from ~47% to ~8%). Percentage of GFP fluorescence area overlapping with Lifeact-RFP was sampled in three different regions of each intestine defined by a 100 x 100 (pixel^2^) box positioned at random (n = 18 per genotype). Analysis of standard deviations was performed by the student’s T-test. Error bars are SEM. Asterisks indicate significant differences in the one-tailed Student’s t-test (*** p< 0.001). Scale bars represent 10 μm.

To determine if the EHBP-1 CH-domain interacts with actin microfilaments or microtubules, we assayed for interaction *in vitro* using co-sedimentation assays (Fig 5A–5F). First, we validated our co-sedimentation assays using human Utrophin actin binding CH-domains (aa1-261) and detected significantly elevated co-sedimentation of this Utrophin fragment with filamentous actin (S7C Fig). Similarly, we found that a purified fusion of GST to the EHBP-1 CH-domain increased its sedimentation by more than 5-fold in the presence of actin microfilaments, indicating that the EHBP-1 CH domain binds to polymerized actin (Fig 5A–5C). By contrast, addition of microtubules to the reaction failed to enhance GST-CH sedimentation (Fig 5D–5F). These results suggested that the EHBP-1 CH-domain functions to link EHBP-1 to polymerized actin.

### Association of EHBP-1 labeled tubular recycling endosomes with actin *in vivo*

Previous work in *C*. *elegans* indicated that microtubules are required for the structure of tubular endosomes in the basolateral intestine [37]. Because we found binding of EHBP-1 to actin microfilaments *in vitro*, we asked whether actin polymerization is also important for the structure of these endosomes. Thus we injected the actin depolymerizing drug latrunculin B (LatB) into the worm pseudocoelom (body cavity) and assayed for effects on the EHBP-1-GFP labeled tubular endosomal meshwork. Indeed, LatB treatment greatly disrupted the EHBP-1-GFP pattern, converting many of the tubules to puncta (Fig 5G–5H and Fig 5J and 5K). Similar treatment with the microtubule-depolymerizing drug nocodazole (Noc) affected the EHBP-1-GFP meshwork in a different manner. The tubular network was still observed, but in a dotted line pattern (Fig 5I–5K). We also assayed the distribution of PH(PLCδ)-GFP labeled basolateral endosomal tubules after LatB and Noc treatments and observed similar results (S5A–S5D Fig). These results indicate that formation or maintenance of basolateral tubular endosomes labeled by EHBP-1 requires both actin and microtubule cytoskeletal elements, although EHBP-1 itself is probably actin-specific in its interactions.

To further test the functional involvement of actin and microtubules in EHBP-1 mediated recycling we assayed Lat B and Noc treatments for effects on the well-defined recycling CIE cargo marker hTAC-GFP [6, 15]. In our previous work we showed that loss of EHBP-1 specifically impaired hTAC-GFP recycling [15]. Our analysis indicates that hTAC-GFP accumulates intracellularly in intestinal epithelial cells after depolymerization of either actin or microtubules (S6A–S6D Fig).

If EHBP-1 links endosomal membranes to actin microfilaments then we would expect to find colocalization of EHBP-1-GFP with F-actin marker Lifeact-RFP. Indeed we found that many punctate regions of endosomes labeled by EHBP-1-GFP in intestinal cells were positive for Lifeact-RFP (Fig 5L–5L"). This is consistent with the localization of actin to endosomes [19]. Likewise, some tubular overlap was observed between EHBP-1-RFP and EMTB-GFP, a marker for microtubules (S6E–S6E" Fig), indicating that EHBP-1 labeled tubular endosomes orient co-linearly with cortical microtubules.

We also analyzed the importance of the EHBP-1 CH domain for EHBP-1 colocalization with actin. We found that CH-GFP, lacking the other domains of EHBP-1, colocalized with Lifeact-RFP on intestinal endosomes (S6F–S6F" Fig). To determine whether the EHBP-1 localization with actin is dependent on its CH domain *in vivo*, we assayed for colocalization of EHBP-1 missing the CH domain with Lifeact-RFP in wild-type and in a *rab-10(ok1494)* mutant background. EHBP-1(ΔCH)-GFP retained some overlap with Lifeact-RFP (Fig 5M–5M" and Fig 5O). However, in *rab-10(ok1494)* mutant animals, EHBP-1(ΔCH)-GFP fusion protein overlap with Lifeact RFP decreased ~83%, with most remaining GFP positive structures offset from Lifeact-RFP puncta (Fig 5N–5N" and Fig 5O). Thus we interpret the EHBP-1(ΔCH)-GFP colocalization with Lifeact-RFP to be mediated via interaction with endogenous RAB-10 and not direct interaction with actin.

### RAB-10(GTP) promotes the interaction of EHBP-1 with actin filaments

Our data suggested that EHBP-1 CH-domain associates with endosomal actin microfilaments *in vitro* and *in vivo*. To determine whether the RAB-10-binding CC-domain influences the actin affinity of the EHBP-1 CH-domain, we assayed for effects of RAB-10 on the ability of a GST-CH-CC fusion protein to co-sediment with F-actin. Without addition of RAB-10 to the reaction, an EHBP-1 fragment containing the CH and CC domains displayed a similar level of interaction with F-actin as the CH domain alone (Fig 5A and S7A and S7B Fig).

**Fig 6:**
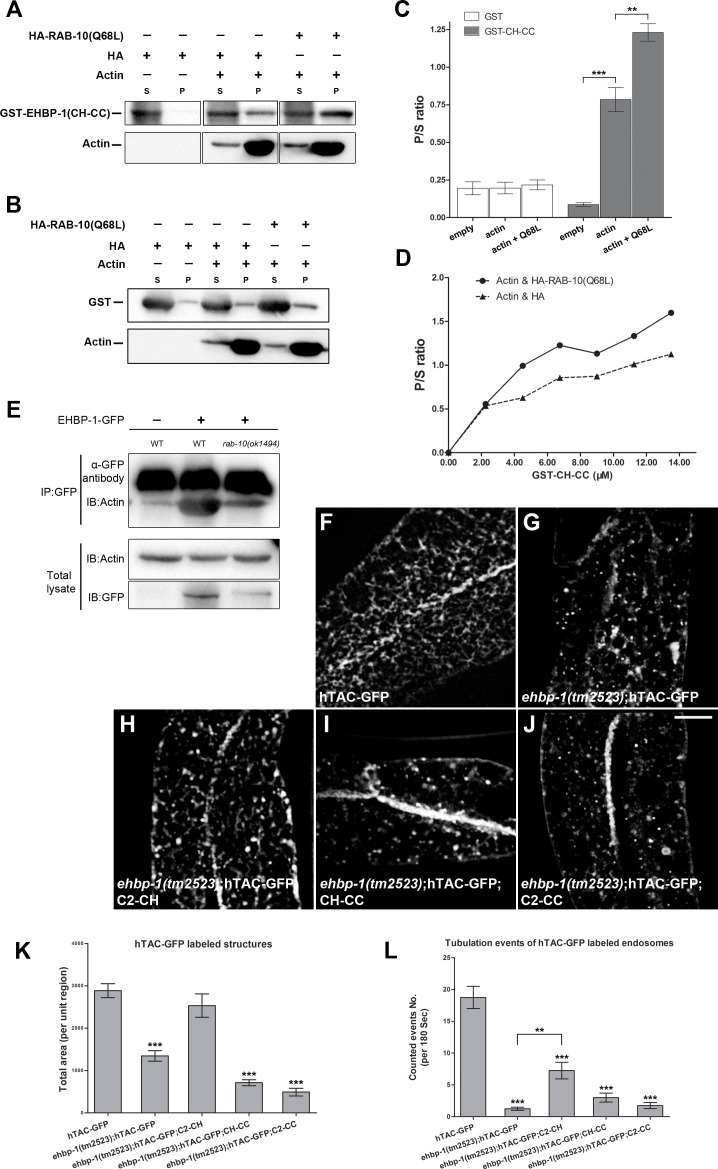
RAB-10(GTP) promotes co-sedimentation of EHBP-1 with F-actin and C2-CH partially restores hTAC-GFP tubular endosomal localization in an *ehbp-1(tm2523)* mutant background. **(A)** GST-CH-CC(260-901aa) co-sediments with actin filaments *in vitro*. The co-sedimentation level of GST-CH-CC with actin filaments increased by ~67% when complexed with HA-RAB-10(Q68L), a predicted constitutively active form of RAB-10. **(B)** control protein GST did not co-sediment with actin filaments. P/S ratio (pellet/supernatant) was quantified for GST-CH-CC and GST in **(C)**, error bars are SEM (n = 3), asterisks indicate significant differences in the one-tailed Student’s t-test, ** p<0.01, *** p<0.001). **(D)** Equilibrium binding of GST-CH-CC to F-actin measured by titrating 21 μM F-actin with 2.25 uM to 13.5 uM GST-CH-CC in the presence of HA-RAB-10(Q68L). **(E)** Co-immunoprecipitation of EHBP-1-GFP and endogenous actin in wild type and *rab-10(ok1494)* animals. EHBP-1-GFP was immunoprecipitated with anti-GFP antibody and precipitants were analyzed by immunoblotting using anti-actin antibody. Aliquots of total lysates (2% of the total input into the assay) were examined by immunoblotting using anti-actin and anti-GFP antibodies. **(F)** In intestinal epithelia hTAC labels basolateral tubular and punctate recycling endosomes. **(G)** In *ehbp-1(tm2523)* mutant animals, hTAC-GFP over-accumulated with a significant loss of hTAC-GFP positive tubules. **(H)** Transgenic expression of the C2-CH fragment partially rescued hTAC-GFP tubular labeling. **(I-J)** Few tubular structures were observed in *ehbp-1(tm2523)* mutant intestinal cells with transgenic expression of CH-CC or C2-CC fragments. Total area (per unit region) was quantified for hTAC-GFP labeled structures in **(K)**, error bars are SEM (n = 18 each, 6 animals of each genotype sampled in three different regions of each intestine defined by a 100 x 100 (pixel^2^) box positioned at random), asterisks indicate significant differences in the one-tailed Student’s t-test *** p<0.001). Scale bar represents 10 μm. **(L)** Tubule movement events were quantified for hTAC-GFP labeled endosomes in WT or *ehbp-1(tm2523)* animals as indicated in **(F-J)**. C2-CH expression presented ~7 movement events per 180 sec, compared with ~18 events in wild-type animals and ~1 event in *ehbp-1(tm2523)* mutant animals. Transgenic expression of CH-CC or C2-CC failed to rescue hTAC-GFP movement defects in *ehbp-1(tm2523)* mutants. Asterisks indicate significant differences in the one-tailed Student’s t-test (**p< 0.01, *** p< 0.001).

However, we detected elevated co-sedimentation of CH-CC with F-actin in the presence of active HA-RAB-10(Q68L), suggesting that RAB-10(GTP) interaction with the EHBP-1 CC-domain enhances the ability of EHBP-1 to bind to actin filaments (Fig 6A and Fig 6C and 6D). In contrast, addition of HA-RAB-10(Q68L) did not enhance the ability of an EHBP-1 fragment lacking the RAB-10 binding CC-domain (C2-CH) to co-sediment with actin (S7D and S7E Fig). As expected, we did not detect interaction between a GST control protein and F-actin (Fig 6B and Fig 6C). Consistent with the *in vitro* data, we found that the EHBP-1(CH-CC)-GFP colocalized well with Lifeact-RFP in the *C*. *elegans* basolateral intestine (S6G–S6G" Fig).

Importantly, the augmented physical interaction between EHBP-1 and F-actin in the presence of RAB-10 was further confirmed *in vivo* by co-immunoprecipitation experiments between GFP-tagged EHBP-1 and endogenous actin in whole-worm lysates. EHBP-1-GFP was immunoprecipitated from worm lysates using an anti-GFP antibody and the precipitants were probed with an anti-actin antibody on western blots. The amount of actin co-immunoprecipitating with EHBP-1-GFP was strongly reduced in *rab-10(ok1494)* mutants, suggesting that RAB-10 promotes the interaction of EHBP-1 with F-actin (Fig 6E).

### EHBP-1 mediated bridging of endosomal membranes and actin microfilaments promotes endosomal tubularity

Loss of EHBP-1 disrupts the tubular endosomal network as visualized by hTAC-GFP (Fig 6F and 6G) or ARF-6-RFP (S8A and S8B Fig). Using the integrity of the hTAC-GFP labeled network as an assay, we sought to test the functionality of versions of EHBP-1 containing different combinations of domains. Importantly we found that overexpression of an EHBP-1 fragment including the membrane associating NT-C2 and F-actin binding CH domains can partially rescue the steady state tubular pattern of recycling cargo marker hTAC-GFP (Fig 6F and 6H and Fig 6K). This was in sharp contrast to the effects of expressing a CH-CC fragment or C2-CC fragment, neither of which could restore hTAC-GFP tubularity (Fig 6I and 6J and Fig 6K). This difference in rescuing ability was even more apparent in time-lapse imaging. Normally the hTAC-GFP labeled endosomal network in the basolateral intestine is highly dynamic, with frequent movement of puncta and tubules (Fig 6L and S1 Video). In *ehbp-1* mutant animals the hTAC-GFP labeled endosomes are devoid of movement, appearing almost completely static (Fig 6L and S2 Video). This could be significantly rescued in an *ehbp-1* mutant expressing the C2-CH fragment, but not upon expression of CH-CC or C2-CC fragments (Fig 6L and S3–S5 Videos). Compared with ~18 tubule movement events (per unit area) /180 sec in wild-type animals, and ~1 event/180 sec in *ehbp-1(tm2523)* mutant animals, C2-CH expression animals presented moderate dynamics with ~7 events/180 sec (Fig 6L). These results are consistent with an important role for EHBP-1 in linking the endosomal membrane to the actin cytoskeleton, and the EHBP-1 CC domain-RAB-10 interaction acting as an enhancer for EHBP-1 CH domain-actin filaments binding during endocytic recycling, regulating membrane tubule formation and function.

## Discussion

Our studies in *C*. *elegans* have demonstrated a requirement for EHBP-1 in basolateral recycling of CIE cargo in intestinal epithelia and postsynaptic recycling of AMPA receptors in interneurons, functioning with the small GTPase RAB-10 [10, 15]. EHBP-1 is enriched in the intestinal cells on basolateral tubular and punctate endosomes, and loss of EHBP-1 results in reduced levels of interacting protein RAB-10 on endosomal membranes [15]. Loss of RAB-10 or EHBP-1 also completely disrupts the tubular character of these endosomes [15]. Our new data suggests that this loss of tubular character, which is closely linked with recycling endosome function, is due to a loss of EHBP-1-dependent linkage between endosomal membranes and F-actin.

The EHBP-1 N-terminal domain was predicted by bioinformatics to adopt a C2 domain-like fold (termed NT-C2) that might allow it to bind to membrane phosphatidylinositols, while the central CH domain suggested an interaction with the cytoskeleton [18, 22]. In this study, we demonstrated the pivotal roles of EHBP-1 NT-C2 domain and CH domain in EHBP-1-mediated recycling regulation. Using *in vitro* and *in vivo* assays, we showed that the NT-C2 domain association with endosomal membranes requires two groups of basic residues predicted to form surface patches that could interact with phosphoinositides. We also found that the CH-domain associates with actin filaments but not microtubules, and that F-actin is important for developing the tubular character of these EHBP-1 associated endosomes. Remarkably, we found that the interaction of the EHBP-1 CC domain with RAB-10(GTP) enhanced the CH domain affinity for actin filaments. Thus our studies suggest that RAB-10 promotes bridging of recycling endosomes and actin filaments via EHBP-1 to create or maintain endosomal tubulation.

Recent phylogenetic analysis and structural modeling predicted an NT-C2 domain in the Ehbp1/EHBP-1 extreme N-terminus, providing a potential interface for EHBP-1 membrane lipid binding [18]. Studies focusing on the well known Ca^2+^-dependent C2 domain of PKC and the Ca^2+^-independent C2 domain of PI3K proposed that C2 domain lipid binding capacity involves two structural segments including a calcium binding pocket-like structure and a β-sandwich surface respectively [41, 42]. The negatively charged acidic residues in the pocket can coordinate Ca^2+^ and lipid binding [41, 43]. The clustered positively charged basic residues (H, R and K) within the β-sandwich regions of Ca^2+^-independent C2 domains participate in the interaction with negatively charged lipids [18]. However, bioinformatics predictions of the membrane binding mode of NT-C2 family proteins suggested that the NT-C2 extreme N-terminus, prior to strand-1, contains a patch of basic residues on the surface, contributing to lipid binding in parallel with the β-sandwich concave surface [18]. Our data revealed two regions of basic residues that appear to contribute to EHBP-1 NT-C2 membrane association. In our structural model these two basic regions appear to be located on opposite sides of the domain. Further structural dissection of the NT-C2 will be required to determine the true arrangement.

As reported in previous studies, PI(4,5)P2 and PI4P are both enriched in recycling endosomes and are important for recycling transport [20, 21]. Our experimental results clearly indicated that the EHBP-1 NT-C2 domain is required for association with tubular endosomes and interacts with PI(4,5)P2. Within the limits of our assays, we did not observe obvious changes in the tubular endosomal network upon knockdown of PI3 kinases known to be important for early endosome function, such as type III PI3-kinase VPS-34 or type I PI3-kinase AGE-1, suggesting that they mainly affect other aspects of endosome function [44–55]. Phosphatidylserine (PS) is also known to be quite important for recruitment of many peripheral membrane proteins necessary for membrane traffic, including endocytic recycling, and phosphatidic acid (PA) has been implicated in recycling tubule formation in mammalian cells [56–61]. It will be important to test for roles of PS and PA in EHBP-1 function in the future.

C2 domains display a wide range of lipid selectivity, with preference for anionic PS and phosphatidylinositol-phosphates (PIPs) [41]. Unlike lipid binding PH domains [62], C2 lipid targeting often involves two recognition components, such as two lipids or a lipid/protein combination. For instance the protein kinase C (PKC) C2 domain uses its basic surface residues to bind plasma membrane PS and PI(4,5)P2 [63], while cytosolic phospholipase A2 (cPLA2) binds to the neutral lipid phosphatidylcholine (PC) and the anionic lipid ceramide-1-phosphate (C1P) through C2 domain Ca^2+^ site charged hydrophobic side chains and a basic cluster [64]. Synaptotagmin utilizes two C2 domains to bridge the vesicular and plasma membranes, with the C2A domain binding vesicular PS and SNARE, while the C2B domain binds plasma membrane PI(4,5)P2 and SNARE [65, 66].

The molecular basis for phosphoinositide-binding specificity of C2 and C2-like domains has been explored in recent years. Structural analysis of the PKCα C2 domain showed that PI(4,5)P2 binds to the concave surface of β3 and β4 strands. Intriguingly, aromatic residues Tyr^195^(strand 2) and Trp^245^(strand 5) interact directly with the inositol ring phosphate moieties of PI(4,5)P2. Loss of Tyr^195^ and Trp^245^ abrogated PI(4,5)P2 recognition and plasma membrane association of PKCα [67]. Phylogenetic analysis showed that Tyr^195^ and Trp^245^ are conserved among different C2 domains except in the DOCK-C2 and NT-C2 families [18]. However, Trp^71^ of EHBP-1/Ehbp1 NT-C2 strand 4 is highly conserved among NT-C2 family members. One plausible possibility is that the Trp^71^ residue participates, at least in part, in PI(4,5)P2 binding specificity. Further functional analysis will be required to test this model.

Filamentous actin has long been known to be particularly important for the Arf6-mediated recycling of CIE cargo such as TAC and MHCI [5, 32]. Colocalization assays and the presence of predicted actin-binding domains have indicated that NT-C2 proteins are involved in actin binding [13, 18]. For instance Ehbp1 colocalizes with cortical actin filaments in cultured mammalian adipocytes and in Drosophila pII cells of the external mechanosensory organs [13]. Since certain CH domains have extensively documented actin binding and bundling functions, we hypothesized that EHBP-1 would link to the actin cytoskeleton via its CH domain [68–70]. Accordingly, our work strongly suggests that EHBP-1 promotes endosomal tubulation by linking PI(4,5)P2 enriched endosomal membranes to F-actin.

Microtubules are also critical players in many different intracellular trafficking processes. Although some CH domains bind to microtubules, *C*. *elegans* tubular endosomes aligned along microtubules, and hTAC recycling in the intestine is impaired upon microtubule disruption, no interaction of the EHBP-1 CH-domain with microtubules was detected in our assays. Thus we infer that while EHBP-1 is microfilament-specific, microtubules play an important role in *C*. *elegans* CIE cargo basolateral recycling, collaborating with the microfilament cytoskeleton to shape the endosomal network [37]. EHBP-1 may promote endosome tubulation by transducing force from growing actin filaments to endosomal membranes. Alternatively, EHBP-1 may anchor endosomal membranes to the actin cytoskeleton while other forces, such as pulling by microtubule motors, acts to deform the membranes.

CH-domain based actin binding structures, such as those found in alpha-actinin and spectrin, often present as a tandem arrangement of two CH domains (CH1-CH2) [22, 71]. The CH1-CH2 dimer takes on a juxtaposed conformation, with weak F-actin affinity until the dimer adopts an open conformation [72, 73]. Utrophin and dystrophin atomic structural models suggest a theme of tandem CH-domains, with one CH domain apposed to the other CH, within the same molecule or provided by two different molecules [74, 75].

In the current study our experiments indicated that the EHBP-1 CH domain mediates the interaction with F-actin, and suggested that the RAB-10 interaction with the EHBP-1 CC-domain somehow potentiates the CH domain-F-actin interaction. Since we did not detect a difference in F-actin binding of the EHBP-1 CH only versus CH-CC fragments, and we also did not detect binding of the CH domain to the CC domain, we do not favor an auto-inhibition model for RAB-10 mediated activation of EHBP-1 actin binding activity (S8C Fig). Rather, since RAB-10 interacts with a predicted coiled-coil domain in EHBP-1, RAB-10 binding may potentiate EHBP-1 multimerization, producing a multivalent presentation of apposed CH domains from the dimerized EHBP-1 molecules. Further analysis will be required to test this model (S8D Fig).

We also note that in mammalian adipocytes Rab10 and Ehbp1 are key regulators of insulin stimulated GLUT4 recycling, but their relationship has not been tested [12, 14, 76]. Future investigation of the Ehbp1-mediated bridging of endosomal membranes and the actin cytoskeleton in human adipocytes could prove fruitful.

## Materials and Methods

### General methods and strains

All *C*. *elegans* strains were derived originally from the wild-type Bristol strain N2. Worm cultures, genetic crosses, and other *C*. *elegans* husbandry were performed according to standard protocols [77]. Strains expressing transgenes were grown at 20°C. A complete list of strains used in this study can be found in S1 Table.

RNAi was performed using the feeding method [78]. Feeding constructs were either from the Ahringer library [79] or prepared by PCR from EST clones provided by Dr Yuji Kohara (National Institute of Genetic, Japan) followed by subcloning into the RNAi vector L4440 [78]. For most experiments, synchronized L1 or L3 stage animals were treated for 48–72 h and were scored as adults.

### Antibodies

The following antibodies were used in this study: rabbit anti-actin polyclonal antibody (sc-1616-R) (Santa Cruz Biotechnologies, Dallas, TX), rabbit anti-HA monoclonal antibody (C29F4) (Cell Signaling Technology, Beverly, MA), rabbit anti-GST monoclonal antibody (91G1) (Cell Signaling Technology, Beverly, MA) and rabbit anti-GFP polyclonal antibody-Chip Grade (ab290) (Abcam, Cambridge, UK).

### Protein expression

N-terminally hemagglutinin (HA)-tagged proteins, 2xHA only and RAB-10(Q68L) were synthesized *in vitro* using the TNT-coupled transcription-translation system (Promega, Madison, WI) using DNA templates pcDNA3.1-2xHA-Gtwy and pcDNA3.1-2xHA-RAB-10(Q68L) (1μg/each 50μl reaction), respectively. The reaction cocktail was incubated at 30°C for 90 min. Control glutathione S-transferase (GST), GST-EHBP-1(260-510aa), GST-EHBP-1(1-510aa), GST-EHBP-1(260-901aa) and GST-hUtrophin actin binding domain (1-261aa) fusion proteins were expressed in the ArcticExpress strain of *Escherichia coli* (Stratagene, La Jolla, CA). Bacterial pellets were lysed in Lysis solution (50 mM HEPES pH 7.5, 400 mM NaCl, 1 mM DTT, 1 mM PMSF or Complete Protease Inhibitor Cocktail Tablets (Roche, Indianapolis, IN)). Extracts were cleared by centrifugation, and supernatants were incubated with glutathione-Sepharose 4B beads (Amersham Pharmacia, Piscataway, NJ) at 4°C overnight. For GST pull down, beads were washed six times with cold STET buffer (10 mM Tris-HCl pH 8.0, 150 mM NaCl, 1 mM EDTA, 0.1% Tween-20). *In vitro* synthesized HA-tagged protein (15 μl TNT mix diluted in 500μl STET) was added to the beads and allowed to bind at 4°C overnight. After six additional washes in STET, the proteins were eluted by boiling in 30μl 2xSDS-PAGE sample buffer. Eluted proteins were separated on SDS-PAGE (12% polyacrylamide), blotted to PVDF, and probed with anti-HA (C29F4) and anti-GST (91G1) antibodies. For protein purification, beads were then washed six times with cold PBS. The bound proteins were eluted with 50 mM Tris-HCL pH 8.0, and 20 mM reduced L-glutathione. Eluted GST fusion peptides were then exchanged into 20 mM HEPES-KOH pH 7.5, 5 mM Mgcl2, 1 mM EGTA, 1 mM DTT. All GST fusion proteins are centrifuged at 150,000x g for 1 h at 4°C prior to use in the co-sedimentation assays at the indicated molar concentrations.

### Liposome co-sedimentation assay

3ug GST-EHBP-1(aa1-223) or GST was mixed with 10ul 1mM Control PolyPIPosomes, PI PolyPIPosomes, PI4P PolyPIPosomes, PI(4,5)P2 PolyPIPosomes, respectively (Echelon Biosciences, Salt Lake City, UT) and rotated for 15 min at room temperature in 1 ml liposome binding buffer (20 mM HEPES pH 7.5, 150 mM NaCl, 1 mM MgCl_2_). The mixture was centrifuged at 90,000xg for 15 min, collecting the supernatant. The liposome pellet was resuspended in 1 ml liposome binding buffer and centrifuged at 90,000xg for 15 min to wash off unspecific bound proteins, this step was repeated three times. The pellet and 20ul supernatant samples were resolved by SDS-PAGE, and GST fusion proteins were detected by western analysis using anti-GST antibody.

### F-actin co-sedimentation assay

Actin co-sedimentation assays were performed using an Actin-Binding Protein Biochem Kit: Non-Muscle Actin (BK013) (Cytoskeleton, Denver, CO), essentially as described by the manufacturer. Supplied α-actinin was used as a positive control. Briefly, protein preparations were incubated with 40ul freshly polymerized non-muscle actin (21 μM F-actin) or F-actin buffer alone. In order to test whether RAB-10(Q68L) enhances GST-EHBP-1(260-901aa) binding to F-actin, *in vitro* synthesized HA-RAB-10(Q68L) and HA-only were added to the mixture. After incubation for 30 minutes at room temperature, samples were centrifuged at 150,000x g for 1.5 h at 24°C to pellet F-actin and the co-sedimenting proteins. Supernatants were collected on ice, and pellets were resuspended on ice for 10 min. SDS-PAGE sample buffer was added to both supernatant and pellet fractions, and the entire fractions were then resolved by SDS-PAGE gel and processed for western blot or stained with coomassie blue. GST-EHBP-1(260-510aa), GST-EHBP-1(260-901aa), GST-EHBP-1(1-510aa) and GST-hUtrophin(1-261aa) co-sedimentations with F-actin were quantified by densitometry using FluorChem FC3 version 3.4.0 (ProteinSimple, San Jose, CA).

### Microtubule co-sedimentation assay

The microtubule-binding assays were performed using the Microtubule Binding Protein Spin-Down Assay Kit (BK029) (Cytoskeleton, Denver, CO). Microtubules were polymerized in cushion buffer (80 mM PIPES pH 7.0, 1 mM MgCl_2_, 1 mM EGTA, 60% glycerol) for 20 min at 35°C and stabilized with taxol. GST-EHBP-1(260-510aa) and the control proteins were mixed separately with microtubules (50μl final volume), incubated at room temperature for 30 min and centrifuged at 100,000x g for 40 min at room temperature on top of a 100 μl of cushion buffer supplemented with taxol. All supernatants and pellets were analyzed by SDS-PAGE as described above.

### Whole-worm immunoprecipitation assay

Worms (9cm plates x 10) were collected and washed with M9 buffer. The worm pellet was lysed by French Press in ice-cold lysis buffer (25 mM Tris-HCl pH 7.5, 100 mM NaCl,1 mM EDTA, 0.5% NP-40, 1 mM PMSF, 1 mM Na_3_VO_4_, 1 μg/ml Pepstatin-A and 10 mM NaF) containing protease-inhibitor cocktail (Sigma, St. Louis, MO). The lysates were incubated at 4°C for 30 min and centrifugated at 13,000xg for 30min. Then, supernatant was incubated with 80μl Protein A+G Agarose (Beyotime, Shanghai, China) for 1h at 4°C to pre-clear non-specific bead-protein interactions. 2μl anti-GFP antibody (ab290) was added into pre-cleared supernatant and incubated at 4°C overnight, followed by incubation with 80μl Protein A+G Agarose (Beyotime, Shanghai, China) at 4°C for 4 hours. Precipitates were washed five times with lysis buffer and subjected to immunoblotting using anti-actin and anti-GFP polyclonal antibodies.

### Plasmids and transgenic strains

*rab-10*(Q68L) cDNA clones were transferred into an in-house modified vector pcDNA3.1(+) (Invitrogen, Carlsbad, CA) with 2xHA epitope tag and Gateway cassette (Invitrogen, Carlsbad, CA) for *in vitro* transcription/translation experiments. For actin binding experiments an equivalent *ehbp-1*(260-510aa), *ehbp-1*(1-510aa), *ehbp-1*(260-901aa) and *hUtrophin*(1-261aa) PCR product was introduced in frame into vector pGEX-2T (GE Healthcare Life Sciences, Piscataway, NJ) modified with a Gateway cassette.

To construct GFP or RFP fusion transgenes for expression specifically in the worm intestine, a previously described *vha-6* promoter-driven vector modified with a Gateway cassette inserted just upstream of the GFP or red fluorescent protein (tagRFP-T) coding region was used. The sequences of *C*. *elegans ehbp-1*(cDNA), *ehbp-1*(aa1-223), *ehbp-1*(aa1-223)(RRLRR6AALAA), *ehbp-1*(aa1-223)(RR6AA), *ehbp-1*(aa1-223)(RR9AA), *ehbp-1*(aa1-223)(KK13AA), *ehbp-1*(aa1-223)(HRRRK46AAAAA), *ehbp-1*(260-510aa), *ehbp-1*(260-901aa) and *ehbp-1*(1-259aa, 511-901aa) lacking a stop codon were cloned individually into entry vector pDONR221 by PCR and BP reaction, and then transferred into intestinal expression vectors by Gateway recombination cloning LR reaction to generate C-terminal fusions [6]. Integrated transgenic lines for all these plasmids were obtained by microinjection or microparticle bombardment.

### Drug interference assay

Nocodazole (50 μg/mL, M1404) (Sigma, St. Louis, MO) or Latrunculin B (10 μM, sc-203318) (Santa Cruz Biotechnologies, Dallas, TX) was injected into the pseudocoelom of young adult worms 2 h before imaging. Drugs were diluted in DMSO and used at a final concentration of 1% DMSO in egg buffer [118 mM NaCl, 48 mM KCl, 2 mM MgCl_2_, 2 mM CaCl_2_, and 25 mM HEPES (pH 7.3)].

### Confocal microscopy and imaging analysis

Live worms were mounted on 2% agarose pads with 10 mM levamisole. Multi-wavelength fluorescence images were obtained using an FLUOVIEW FV1000 microscope (Olympus, Tokyo, Japan) and captured using FV10-ASW Ver.3.1 software. Images taken in the DAPI channel were used to identify broad-spectrum intestinal autofluorescence caused by lipofuscin-positive lysosome-like organelles. Fluorescence images were obtained using an FV1000-IX81 confocal laser scanning microscope (Olympus, Tokyo, Japan) equipped with a 60×N.A. 1.2 oil-immersion objective. Z series of optical sections were acquired using a 0.5μm step size.

Dynamic fluorescence imaging was performed on a spinning-disk confocal imaging system (CSU-X1) (Yokogawa, Tokyo, Japan) equipped with an EM CCD camera (iXon DU897K) (ANDOR, Belfast, UK)) and oil-immersion objectives (60×N.A. 1.45). A 50 mW solid state lasers (491 nm) coupled to an acoustic-optical tunable filter (AOTF) were used to excite GFP. hTAC-GFP labeled endosomes dynamic images were obtained over 180–240 sec with an exposure every 1 sec.

To compare the subcellular distribution of GFP-tagged proteins, fluorescence data from GFP channel were analyzed by Metamorph software version 7.8.0.0 (Universal Imaging, West Chester, PA). The “Integrated Morphometry Analysis” function of Metamorph was used to detect the fluorescent structures that are significantly brighter than the background and to measure total puncta number (referred as “structure count”) and total fluorescence area (referred as “total area”) within unit regions. From total 6 animals of each genotype, “structure count” and “total area” were sampled in three different unit regions of each intestine defined by a 100 x 100 (pixel^2^) box positioned at random (n = 18 each). In most cases, “total area” was used to compare tubularity, as the normal endosomal tubule network covers much more area than when the network collapses into puncta. Another parameter “structure count” was also sometimes used to assay this aspect, where the structure count increases as the network breaks down into puncta.

GFP and RFP-tagged proteins colocalization analysis were performed using “Measure colocalization” App of Metamorph software. After thresholding, the percentage of GFP fluorescence area (area A) overlapping with RFP fluorescence area (area B) in eighteen intestinal unit regions (3 regions per animal) was analyzed for each genotype. Most GFP/RFP colocalization experiments were performed on L3 and L4 larvae expressing GFP and RFP markers.

To establish a quantitative index for the vacuole phenotype, total vacuole number and size were quantified in three intestinal cells of 6 *ehbp-1(tm2523)* mutant or rescue animals (n = 18 per genotype) using endosome visualization marker ARF-6-RFP. Vacuoles were classified into 3 different size groups: small vacuole (diameter <5 μm), medium vacuole (diameter 5–10 μm) and large vacuole (diameter >10 μm).

## Supporting Information

S1 Fig EHBP-1 lacking N-terminal C2-like or central CH domain failed to rescue the intestinal vacuole (enlarged endosome) phenotype. **(A)** In *ehbp-1(tm2523)* intestinal cells, abnormally enlarged vacuoles labeled by ARF-6-RFP can be observed. **(B-B'')** The vacuole phenotype can be rescued by intestine-specific expression of EHBP-1-GFP. **(C-C'')** Many small and medium size vacuoles can be observed in animals expressing EHBP-1 lacking the NT-C2 domain. **(D-D'')** Transgenic expression of EHBP-1 lacking the CH domain failed to rescue the vacuole phenotype. Instead, more small and medium size vacuoles were observed. Arrows indicate vacuoles in the intestinal cells labeled by ARF-6-RFP. Number of vacuoles with different diameters were quantified and plotted in **(E)**.
(TIF)

S2 Fig RAB-10 differentially influences the localization of EHBP-1 forms lacking NT-C2 or CH domains. **(A)** EHBP-1(ΔNT-C2)-GFP is enriched on basolateral punctate structures in *C*. *elegans* intestinal epithelial cells. **(B)** Puncta localization of EHBP-1(ΔNT-C2)-GFP relies on the presence of RAB-10. In *rab-10(ok1494)* mutant animals EHBP-1(ΔNT-C2)-GFP lost punctate labeling and appeared diffuse in the cytoplasm. **(C)** EHBP-1(ΔCH)-GFP still labels tubular endosomal networks. **(D)** In *rab-10(ok1494)* knockout animals EHBP-1(ΔCH)-GFP accumulates on medial endosomes and the limiting membrane of vacuoles. Arrowheads indicate EHBP-1(ΔNT-C2)-GFP and EHBP-1(ΔCH)-GFP labeled puncta in the intestinal cells. Arrows indicate EHBP-1(ΔCH)-GFP labeled intestinal vacuoles. **(E-F')** Intestinal expression of CH-CC fragment (EHBP-1(ΔNT-C2)) disrupted recycling cargo hTAC-GFP tubular endosomal localization. **(G-G')** hTAC-GFP lost tubular endosomal localization and accumulated on punctate structures upon the knockdown of PPK-1. **(H-I)** Expression of CH-CC fragment caused intracellular accumulation of recycling cargo hTAC-GFP on enlarged endosomes and vacuoles. Arrows indicate hTAC-GFP labeled intestinal vacuoles. Scale bars represent 10 μm.
(TIF)

S3 Fig Association of EHBP-1 fragments with RAB-10 and ARF-6 labeled endosomes. Colocalization images are from confocal image stacks acquired in intestinal epithelial cells of intact living animals. **(A-A")** EHBP-1(NT-C2)-GFP colocalizes with recycling endosome marker ARF-6-RFP on punctate structures. **(B-B")** EHBP-1(NT-C2)-GFP also colocalizes on punctate endosomes with RFP-RAB-10. **(C-C")** EHBP-1(CH)-GFP colocalizes with ARF-6-RFP on endosomal puncta. **(D-D")** EHBP-1(CH)-GFP displayed colocalization with RFP-RAB-10 on basolateral endosomes. **(E-E")** ARF-6-RFP colocalizes with EHBP-1(CC)-GFP on basolateral puncta. **(F-F")** RAB-10 colocalizes well with EHBP-1(CC)-GFP on medial puncta. Scale bars represent 10 μm.
(TIF)

S4 Fig The EHBP-1 NT-C2 domain is not associated with PI(3)P enriched membranes in the *C*. *elegans* intestine. **(A-B")** Colocalization images from intact living animals are presented. EHBP-1(NT-C2)-GFP did not colocalize with PI(3)P biosensor RFP-2xFYVE in *C*. *elegans* intestinal cells. **(C)** Liposome co-sedimentation assay was performed in the presence of liposomes containing 0% PI (Control), 5% PI, 5% PI(4)P or 5% PI(4,5)P2. Liposomes were incubated with 3ug GST as indicated. **(D-E)** Vacuole phenotype cannot be rescued by expression of EHBP-1-GFP containing NT-C2 domain basic motif mutations. Scale bars represent 10 μm.
(TIF)

S5 Fig PH(PLCδ)-GFP labeled basolateral endosomal tubules requires intact F-actin and microtubule cytoskeletons. **(A-A')** PH(PLCδ)-GFP labels tubular endosomes after injection of control DMSO. **(B-B')** After LatB treatment, PH(PLCδ)-GFP labeled tubular meshwork was disrupted, and PH(PLCδ)-GFP puncta number increased by ~34%. **(C-C')** Nocodazole (Noc) treatment also disrupted the PH(PLCδ)-GFP labeled tubular network. **(D)** PH(PLCδ)-GFP labeled puncta number (structure count) within unit region was quantified. Error bars are SEM (n = 18, 6 animals of each treatment were sampled in three different unit regions of each intestine defined by a 100 x 100 (pixel^2^) box positioned at random). Asterisks indicate significant differences in the one-tailed Student’s t-test (**p < 0.01). Scale bars represent 10 μm.
(TIF)

S6 Fig Recycling transport of hTAC-GFP via tubular endosomal networks requires the cytoskeleton. **(A)** In animals injected with DMSO, hTAC-GFP mainly localized to tubular and punctate endosomes. **(B)** After treatment with G-actin sequestering agent latrunculin B (LatB), hTAC-GFP accumulated in enlarged medial structures. The hTAC-GFP labeled tubular network was disrupted and hTAC-GFP positive structure number decreased significantly (~45%). **(C)** Microtubule-depolymerizing drug nocodazole (Noc) treatment also disrupted the hTAC-GFP labeled tubular network and caused accumulation of hTAC-GFP. **(D)** Total fluorescence area of hTAC-GFP signal within unit region was quantified. Error bars are SEM (n = 18 each, 6 animals of each treatment sampled in three different regions of each intestine defined by a 100 x 100 (pixel^2^) box positioned at random). Asterisks indicate significant differences in the one-tailed Student’s t-test (**p< 0.01, *** p< 0.001). Scale bar represents 10 μm. **(E-E")** EHBP-1-RFP and EMTB-GFP partially overlap on tubular and punctate structures. **(F-F")** EHBP-1(CH)-GFP colocalizes with actin marker Lifeact-RFP on sparse medial puncta. Arrows indicate endosomes labeled by both EHBP-1(CH)-GFP and Lifeact-RFP. **(G-G")** EHBP-1(CH-CC)-GFP overlaps well with Lifeact-RFP on basolateral punctate structures. Arrowheads indicate endosomes labeled by both EHBP-1(CH-CC)-GFP and Lifeact-RFP. Scale bars represent 10 μm.
(TIF)

S7 Fig The CH-CC fragment has a comparable level of actin filament co-sedimentation to the CH domain. **(A)** Compared with GST-CH in Fig 5A–5C, GST-CH-CC displayed a similar actin filament co-sedimentation level. P/S ratio (pellet/supernatant) was quantified in **(B)**. Samples were analyzed by SDS-PAGE and coomassie blue stain. **(C)** The hUtrophin actin binding domain (aa1-261) co-sediments with actin filaments *in vitro*. GST-hUtrophin(aa1-261) fusion protein sedimentation percentage shifted significantly in the presence of actin filaments (coomassie blue stained gel). **(D-E)** GST-C2-CH(aa1-510) co-sediments with actin filaments *in vitro*. The co-sedimentation level of GST-C2-CH with actin filaments was not affected when complexed with HA-RAB-10(Q68L). P/S ratio (pellet/supernatant) was quantified for GST-C2-CH in **(E)**, error bars are SEM (n = 3), asterisks indicate significant differences in the one-tailed Student’s t-test, *** p<0.001).
(TIF)

S8 Fig EHBP-1 functional model. **(A)** ARF-6-RFP labels basolateral tubular endosomes. **(B)** In *ehbp-1(tm2523)* mutant animals, the ARF-6-RFP labeled tubular meshwork was disrupted. Arrowheads indicate tubular endosomes labeled by ARF-6-RFP, arrows indicate ARF-6-RFP positive punctate endosomes. Scale bars represent 10 μm. **(C)** An interaction between EHBP-1-CH and CC domains was not detected *in vitro*. Glutathione beads loaded with recombinant GST or GST-EHBP-1-CH (aa260-510) were incubated with *in vitro* expressed HA-tagged EHBP-1-CC (aa510-901), and then washed to remove unbound proteins. Bound proteins were eluted and analyzed by western blot using anti-HA (top) and anti-GST (bottom). Input lanes contain *in vitro* expressed HA-tagged EHBP-1-CC (aa510-901) used in the binding assays (5% and 10%). **(D)** A model for EHBP-1 function. RAB-10 interaction with the EHBP-1 CC-domain potentiates EHBP-1 interaction with F-actin, promoting tubulation and function of recycling endosomes carrying CIE cargo.
(TIF)

S1 Video hTAC-GFP labeled endosome dynamics in wild-type. Example video of a young adult animal showing 1 frame/sec over a 3 min time period.
(AVI)

S2 Video hTAC-GFP labeled endosome dynamics in *ehbp-1(tm2523)* mutant animal. Example video of a young adult animal showing 1 frame/sec over a 3 min time period.
(AVI)

S3 Video hTAC-GFP labeled endosome dynamics in *ehbp-1(tm2523)* mutant animal expressing the EHBP-1 C2-CH fragment. Example video of a young adult animal showing 1 frame/sec over a 3 min time period.
(AVI)

S4 Video hTAC-GFP labeled endosome dynamics in *ehbp-1(tm2523)* mutant animal expressing the EHBP-1 CH-CC fragment. Example video of a young adult animal showing 1 frame/sec over a 3 min time period.
(AVI)

S5 Video hTAC-GFP labeled endosome dynamics in *ehbp-1(tm2523)* mutant animal expressing the EHBP-1 C2-CC fragment. Example video of a young adult animal showing 1 frame/sec over a 3 min time period.
(AVI)

S1 Table Strain list. Summary of the transgenic and mutant strains used in this study.
(DOCX)
