# Supplementary figures and images for: RAB-10 Promotes EHBP-1 Bridging of Filamentous Actin and Tubular Recycling Endosomes
Source: PLoS Genet. 2016 Jun 6;12(6):e1006093. doi: 10.1371/journal.pgen.1006093 (PMC4894640; doi:10.1371/journal.pgen.1006093)

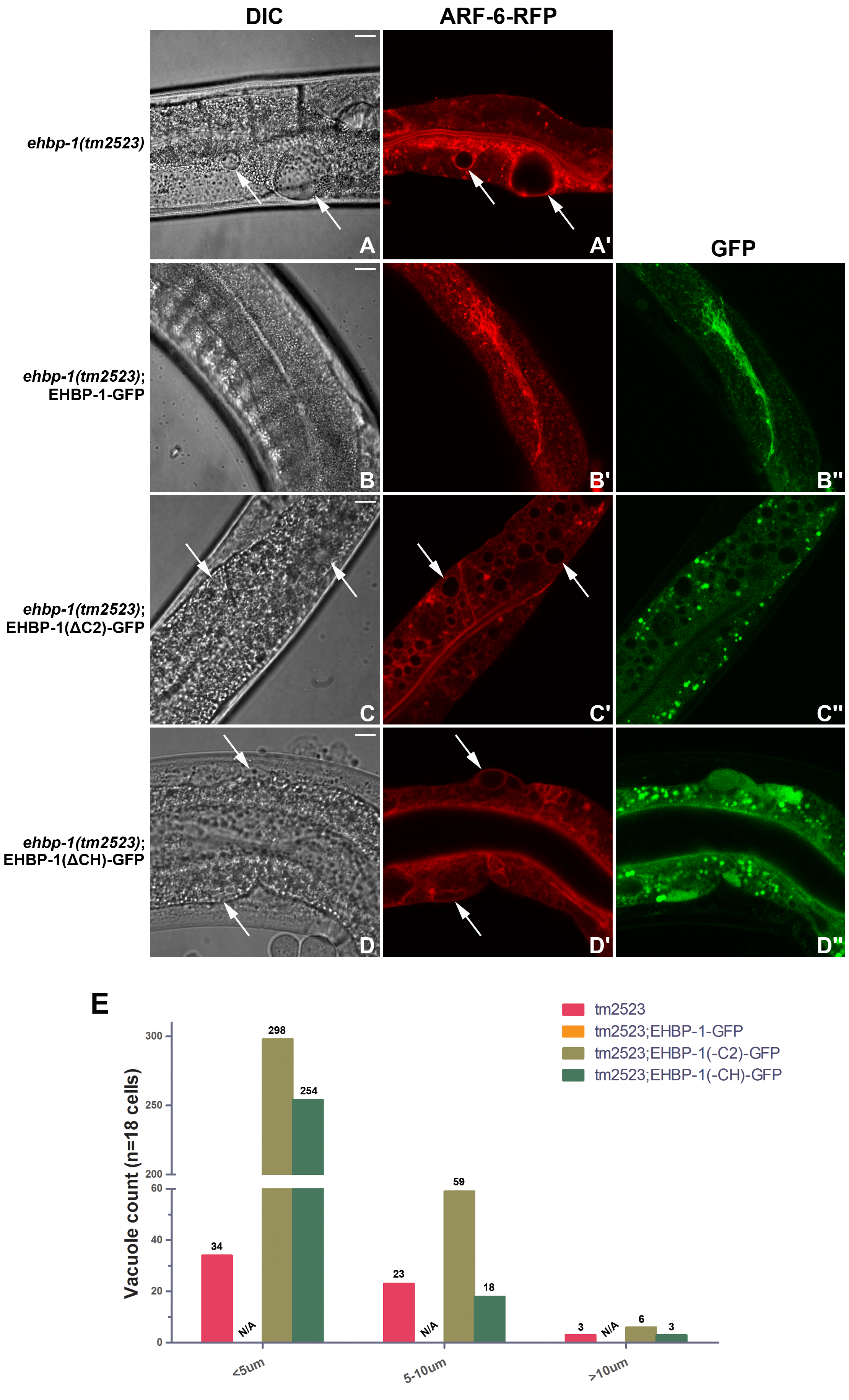

Supplement: S1 Fig — (A) In ehbp-1(tm2523) intestinal cells, abnormally enlarged vacuoles labeled by ARF-6-RFP can be observed. (B-B'') The vacuole phenotype can be rescued by intestine-specific expression of EHBP-1-GFP. (C-C'') Many small and medium size vacuoles can be observed in animals expressing EHBP-1 lacking the NT-C2 domain. (D-D'') Transgenic expression of EHBP-1 lacking the CH domain failed to rescue the vacuole phenotype. Instead, more small and medium size vacuoles were observed. Arrows indicate vacuoles in the intestinal cells labeled by ARF-6-RFP. Number of vacuoles with different diameters were quantified and plotted in (E). (TIF) [file pgen.1006093.s001.tif]

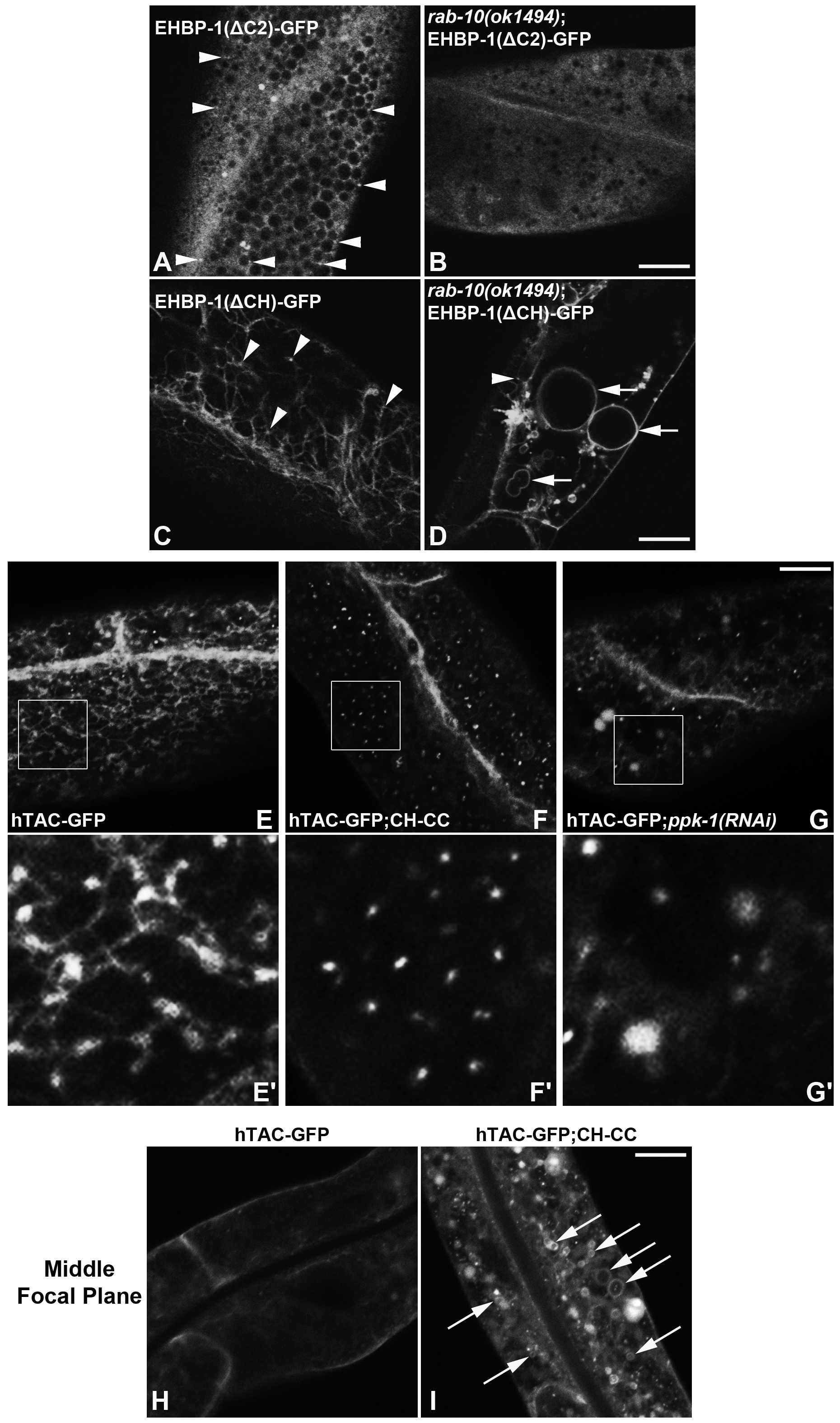

Supplement: S2 Fig — (A) EHBP-1(ΔNT-C2)-GFP is enriched on basolateral punctate structures in C. elegans intestinal epithelial cells. (B) Puncta localization of EHBP-1(ΔNT-C2)-GFP relies on the presence of RAB-10. In rab-10(ok1494) mutant animals EHBP-1(ΔNT-C2)-GFP lost punctate labeling and appeared diffuse in the cytoplasm. (C) EHBP-1(ΔCH)-GFP still labels tubular endosomal networks. (D) In rab-10(ok1494) knockout animals EHBP-1(ΔCH)-GFP accumulates on medial endosomes and the limiting membrane of vacuoles. Arrowheads indicate EHBP-1(ΔNT-C2)-GFP and EHBP-1(ΔCH)-GFP labeled puncta in the intestinal cells. Arrows indicate EHBP-1(ΔCH)-GFP labeled intestinal vacuoles. (E-F') Intestinal expression of CH-CC fragment (EHBP-1(ΔNT-C2)) disrupted recycling cargo hTAC-GFP tubular endosomal localization. (G-G') hTAC-GFP lost tubular endosomal localization and accumulated on punctate structures upon the knockdown of PPK-1. (H-I) Expression of CH-CC fragment caused intracellular accumulation of recycling cargo hTAC-GFP on enlarged endosomes and vacuoles. Arrows indicate hTAC-GFP labeled intestinal vacuoles. Scale bars represent 10 μm. (TIF) [file pgen.1006093.s002.tif]

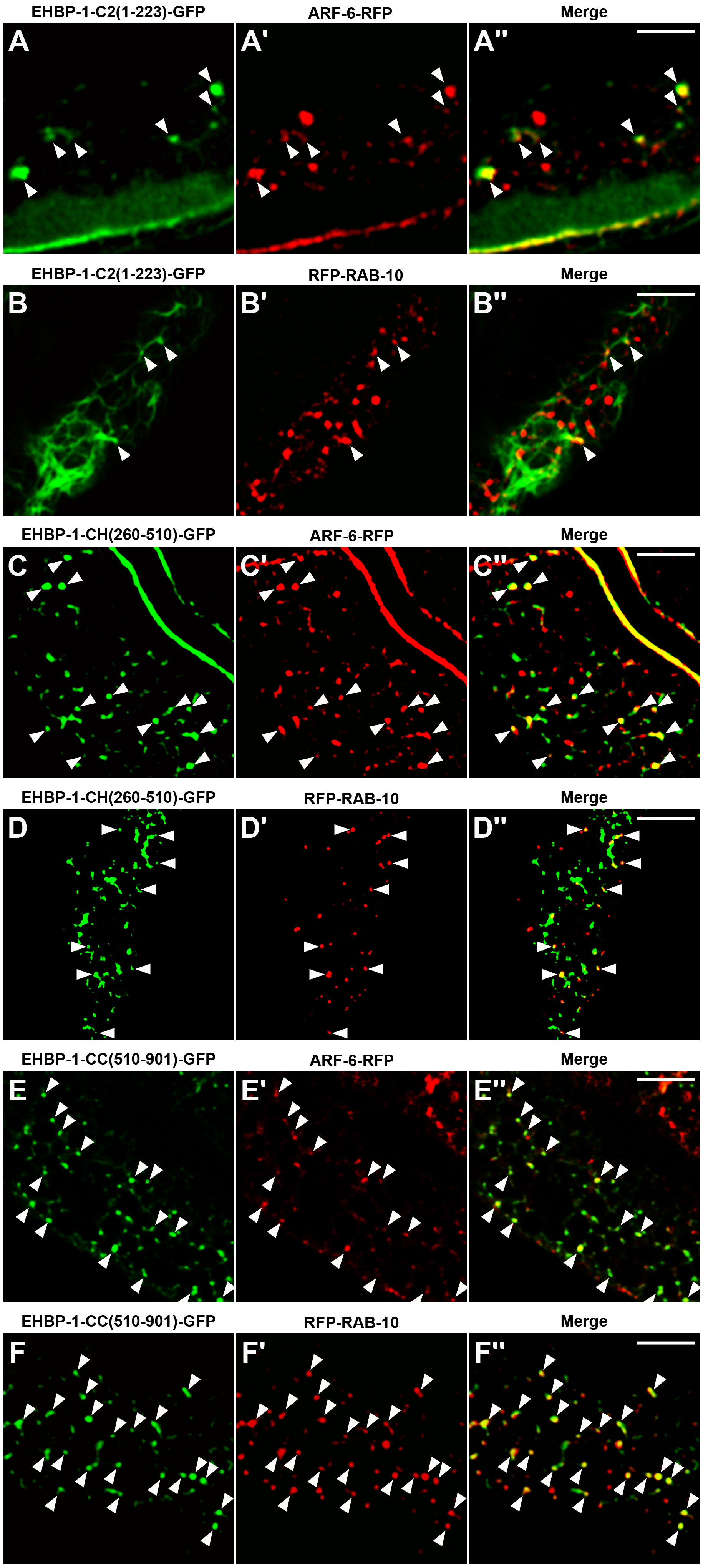

Supplement: S3 Fig — Colocalization images are from confocal image stacks acquired in intestinal epithelial cells of intact living animals. (A-A") EHBP-1(NT-C2)-GFP colocalizes with recycling endosome marker ARF-6-RFP on punctate structures. (B-B") EHBP-1(NT-C2)-GFP also colocalizes on punctate endosomes with RFP-RAB-10. (C-C") EHBP-1(CH)-GFP colocalizes with ARF-6-RFP on endosomal puncta. (D-D") EHBP-1(CH)-GFP displayed colocalization with RFP-RAB-10 on basolateral endosomes. (E-E") ARF-6-RFP colocalizes with EHBP-1(CC)-GFP on basolateral puncta. (F-F") RAB-10 colocalizes well with EHBP-1(CC)-GFP on medial puncta. Scale bars represent 10 μm. (TIF) [file pgen.1006093.s003.tif]

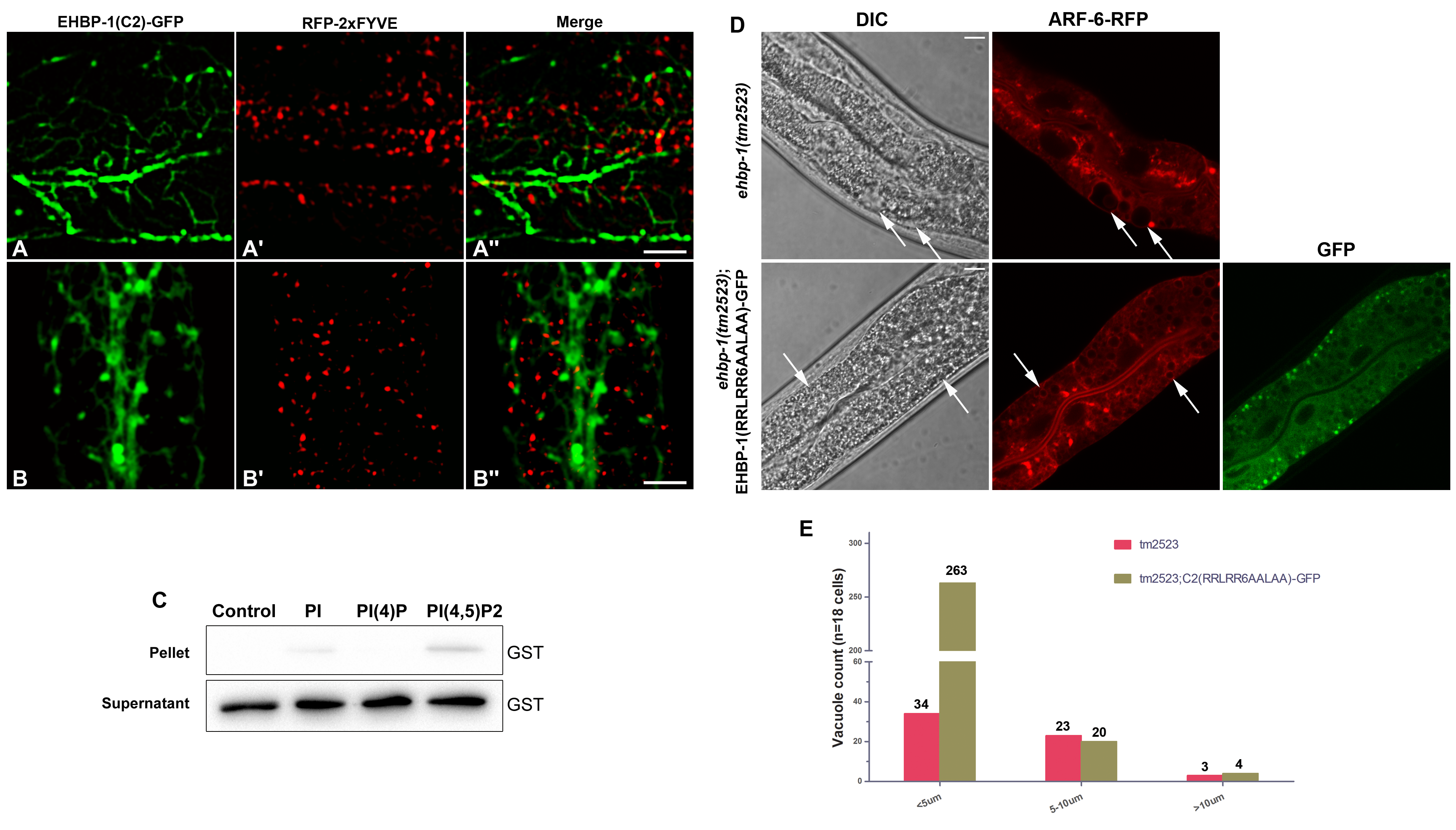

Supplement: S4 Fig — (A-B") Colocalization images from intact living animals are presented. EHBP-1(NT-C2)-GFP did not colocalize with PI(3)P biosensor RFP-2xFYVE in C. elegans intestinal cells. (C) Liposome co-sedimentation assay was performed in the presence of liposomes containing 0% PI (Control), 5% PI, 5% PI(4)P or 5% PI(4,5)P2. Liposomes were incubated with 3ug GST as indicated. (D-E) Vacuole phenotype cannot be rescued by expression of EHBP-1-GFP containing NT-C2 domain basic motif mutations. Scale bars represent 10 μm. (TIF) [file pgen.1006093.s004.tif]

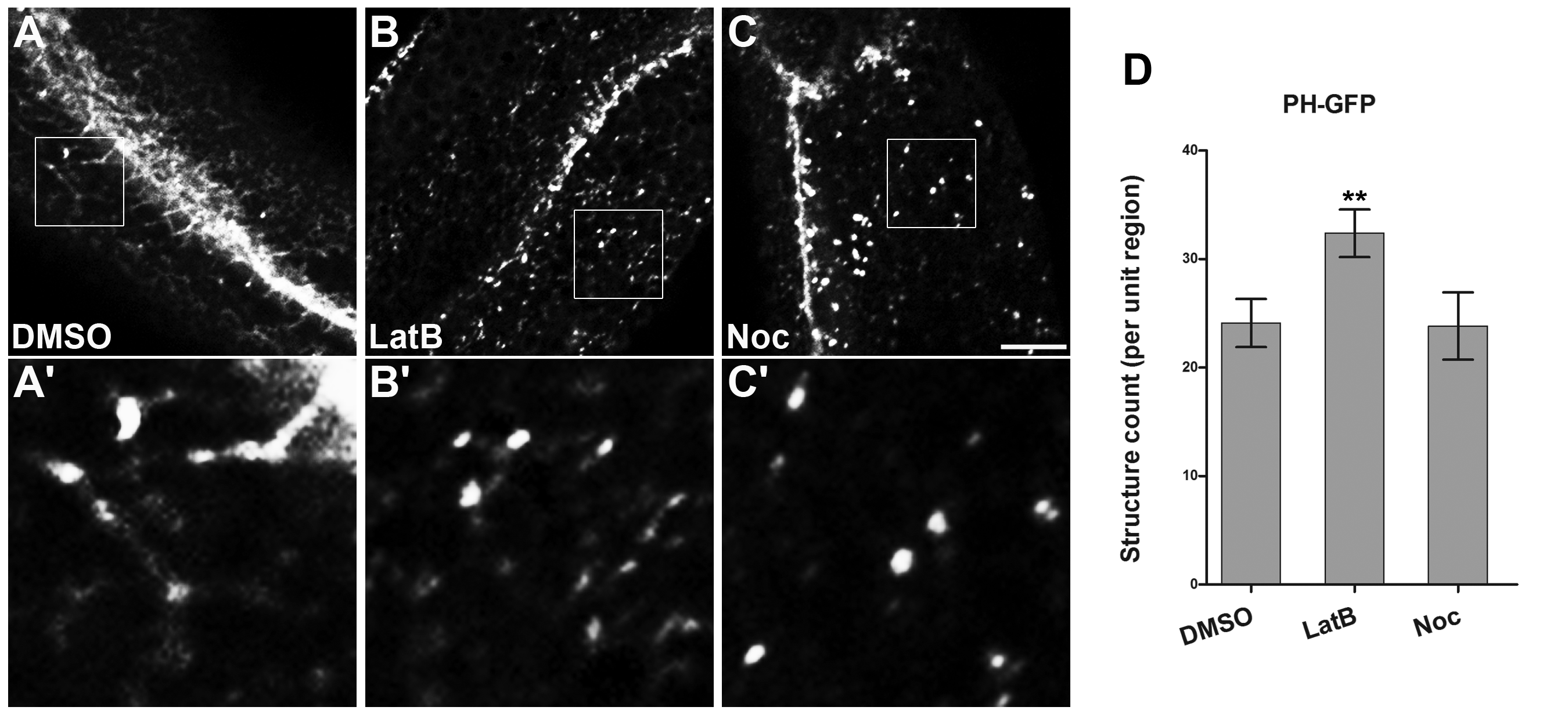

Supplement: S5 Fig — (A-A') PH(PLCδ)-GFP labels tubular endosomes after injection of control DMSO. (B-B') After LatB treatment, PH(PLCδ)-GFP labeled tubular meshwork was disrupted, and PH(PLCδ)-GFP puncta number increased by ~34%. (C-C') Nocodazole (Noc) treatment also disrupted the PH(PLCδ)-GFP labeled tubular network. (D) PH(PLCδ)-GFP labeled puncta number (structure count) within unit region was quantified. Error bars are SEM (n = 18, 6 animals of each treatment were sampled in three different unit regions of each intestine defined by a 100 x 100 (pixel2) box positioned at random). Asterisks indicate significant differences in the one-tailed Student’s t-test (**p < 0.01). Scale bars represent 10 μm. (TIF) [file pgen.1006093.s005.tif]

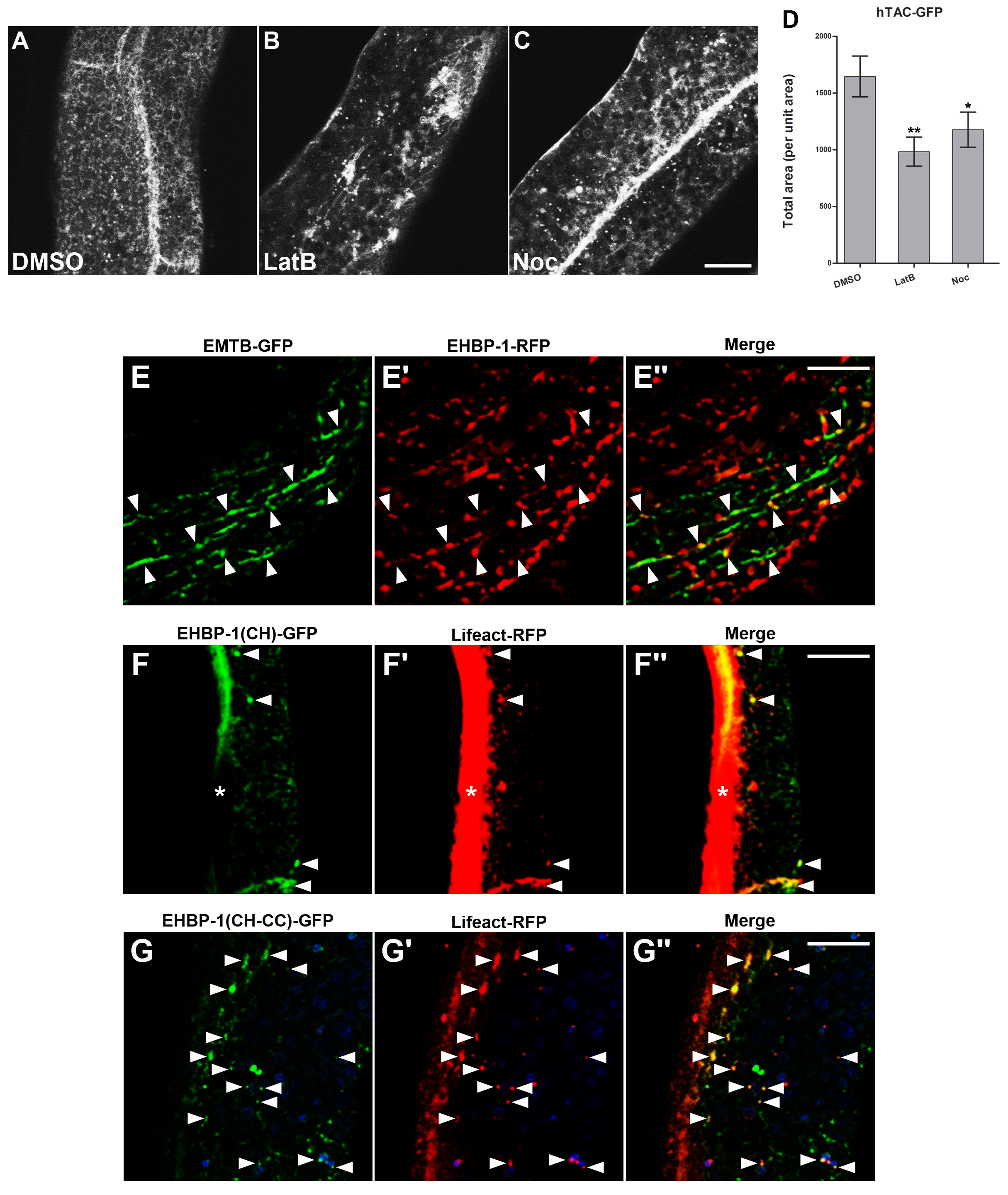

Supplement: S6 Fig — (A) In animals injected with DMSO, hTAC-GFP mainly localized to tubular and punctate endosomes. (B) After treatment with G-actin sequestering agent latrunculin B (LatB), hTAC-GFP accumulated in enlarged medial structures. The hTAC-GFP labeled tubular network was disrupted and hTAC-GFP positive structure number decreased significantly (~45%). (C) Microtubule-depolymerizing drug nocodazole (Noc) treatment also disrupted the hTAC-GFP labeled tubular network and caused accumulation of hTAC-GFP. (D) Total fluorescence area of hTAC-GFP signal within unit region was quantified. Error bars are SEM (n = 18 each, 6 animals of each treatment sampled in three different regions of each intestine defined by a 100 x 100 (pixel2) box positioned at random). Asterisks indicate significant differences in the one-tailed Student’s t-test (**p< 0.01, *** p< 0.001). Scale bar represents 10 μm. (E-E") EHBP-1-RFP and EMTB-GFP partially overlap on tubular and punctate structures. (F-F") EHBP-1(CH)-GFP colocalizes with actin marker Lifeact-RFP on sparse medial puncta. Arrows indicate endosomes labeled by both EHBP-1(CH)-GFP and Lifeact-RFP. (G-G") EHBP-1(CH-CC)-GFP overlaps well with Lifeact-RFP on basolateral punctate structures. Arrowheads indicate endosomes labeled by both EHBP-1(CH-CC)-GFP and Lifeact-RFP. Scale bars represent 10 μm. (TIF) [file pgen.1006093.s006.tif]

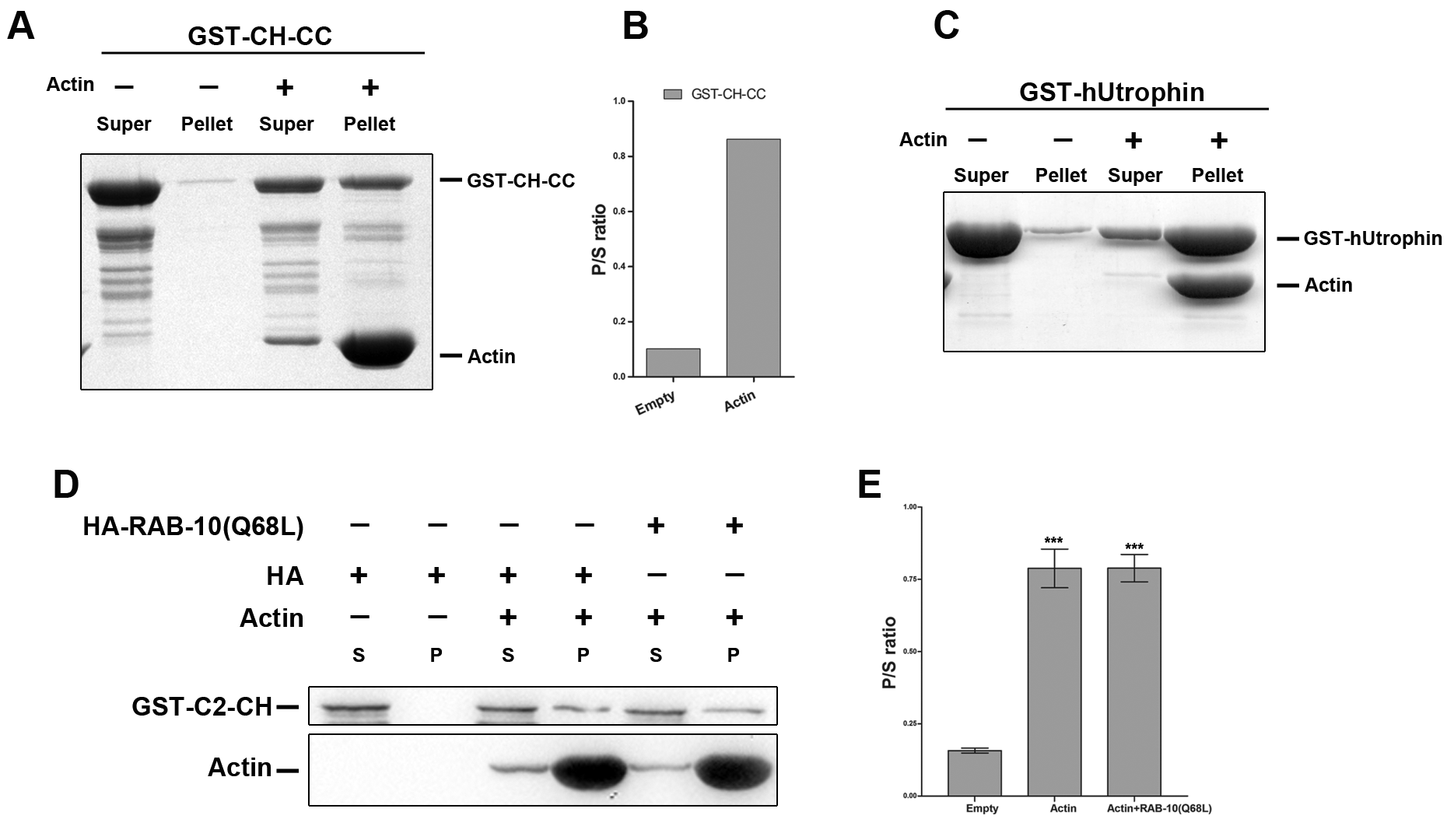

Supplement: S7 Fig — (A) Compared with GST-CH in Fig 5A–5C, GST-CH-CC displayed a similar actin filament co-sedimentation level. P/S ratio (pellet/supernatant) was quantified in (B). Samples were analyzed by SDS-PAGE and coomassie blue stain. (C) The hUtrophin actin binding domain (aa1-261) co-sediments with actin filaments in vitro. GST-hUtrophin(aa1-261) fusion protein sedimentation percentage shifted significantly in the presence of actin filaments (coomassie blue stained gel). (D-E) GST-C2-CH(aa1-510) co-sediments with actin filaments in vitro. The co-sedimentation level of GST-C2-CH with actin filaments was not affected when complexed with HA-RAB-10(Q68L). P/S ratio (pellet/supernatant) was quantified for GST-C2-CH in (E), error bars are SEM (n = 3), asterisks indicate significant differences in the one-tailed Student’s t-test, *** p<0.001). (TIF) [file pgen.1006093.s007.tif]

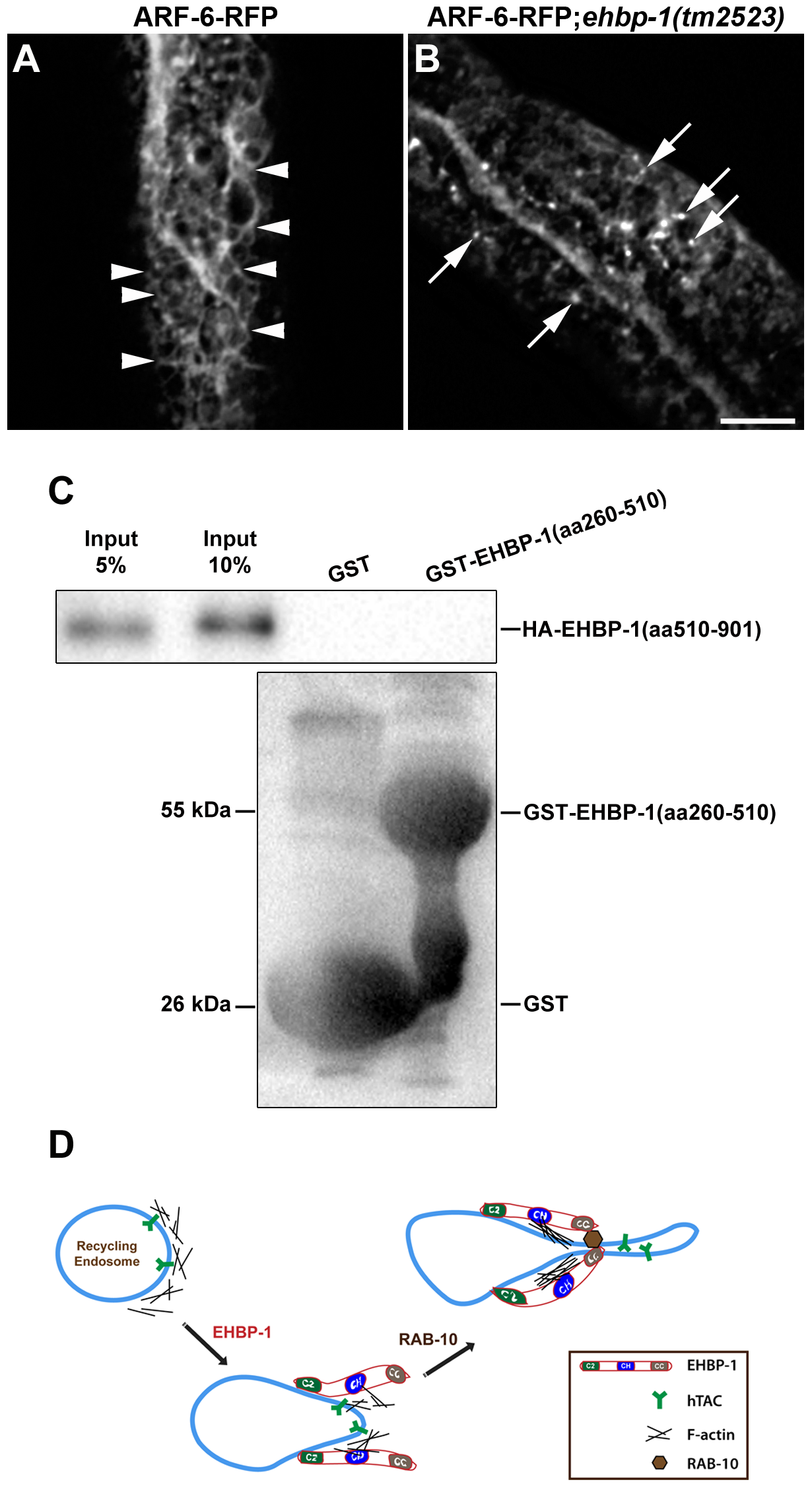

Supplement: S8 Fig — (A) ARF-6-RFP labels basolateral tubular endosomes. (B) In ehbp-1(tm2523) mutant animals, the ARF-6-RFP labeled tubular meshwork was disrupted. Arrowheads indicate tubular endosomes labeled by ARF-6-RFP, arrows indicate ARF-6-RFP positive punctate endosomes. Scale bars represent 10 μm. (C) An interaction between EHBP-1-CH and CC domains was not detected in vitro. Glutathione beads loaded with recombinant GST or GST-EHBP-1-CH (aa260-510) were incubated with in vitro expressed HA-tagged EHBP-1-CC (aa510-901), and then washed to remove unbound proteins. Bound proteins were eluted and analyzed by western blot using anti-HA (top) and anti-GST (bottom). Input lanes contain in vitro expressed HA-tagged EHBP-1-CC (aa510-901) used in the binding assays (5% and 10%). (D) A model for EHBP-1 function. RAB-10 interaction with the EHBP-1 CC-domain potentiates EHBP-1 interaction with F-actin, promoting tubulation and function of recycling endosomes carrying CIE cargo. (TIF) [file pgen.1006093.s008.tif]
