# Supplementary material for: RAB-10 Promotes EHBP-1 Bridging of Filamentous Actin and Tubular Recycling Endosomes
Source: PLoS Genet. 2016 Jun 6;12(6):e1006093. doi: 10.1371/journal.pgen.1006093 (PMC4894640; doi:10.1371/journal.pgen.1006093)
Supplement: S1 Table — Summary of the transgenic and mutant strains used in this study. (DOCX) [file pgen.1006093.s014.docx]

**Table S1**

| Transgenic and Mutant Strains Used in This Study |
| --- |
| *ycxIs12[pvha6::EHBP-1::GFP]*  *ycxIs6[pvha6::EHBP-1(aa157-901)::GFP]*  *ycxEx138[pvha6::EHBP-1(aa1-259_511-901)::GFP]*  *ycxIs1[pvha6::EHBP-1(aa1-223)::GFP]*  *ycxEx58[pvha6::EHBP-1(aa260-510)::GFP]*  *ycxEx135[pvha6::EHBP-1(aa510-901)::GFP]*  *ycxEx7[pvha6::EHBP-1(aa1-223) (RRLRR6AALAA)::GFP]*  *ycxEx50[pvha6::EHBP-1(aa1-223) (HRRRK46AAAAA)::GFP]*  *ycxEx22[pvha6::EHBP-1(aa1-223) (KK13AA)::GFP]*  *ycxEx51[pvha6::EHBP-1(aa1-223) (RR6AA)::GFP]*  *ycxEx32[pvha6::EHBP-1(aa1-223) (RR9AA)::GFP]*  *ycxEx242[Pvha-6::EMTB::3xGFP]*  *ycxEx285[pvha6::Lifeact::tagRFP]*  *ycxEx85[pvha6::EHBP-1(aa1-223)::mCherry]*  *ycxEx286 [pvha6::EHBP-1(1-510aa)::mCherry]*  *ycxEx289 [pvha6::EHBP-1(157-901aa)::mCherry]*  *ycxEx287[pvha6::EHBP-1(1-259_511-901aa)::mCherry]*  *ycxEx288[pvha6::mCherry::2xFYVE]*  *pwIs846 [pvha6::TagRFP::RAB-5][15]*  *pwIs414[pvha6::RFP::RAB-10][15]*  *pwIs625[pvha6::ARF-6::mCherry][20]*  *pwIs112[pvha6::hTAC::GFP][6]*  *pwIs446[pvha6::PH::GFP][20]*  *rab-10(ok1494) (From C. elegans Gene Knockout Consortium)*  *ehbp-1(tm2523) (Dr. Shohei Mitani, Japanese National Bioresource Project for the Experimental Animal“Nematode C. elegans”)*  *arf-6(tm1447) (Dr. Shohei Mitani, Japanese National Bioresource Project for the Experimental Animal“Nematode C. elegans”)* |
